# Impact of climate change on women mental health in rural hinterland of Pakistan

**DOI:** 10.3389/fpsyt.2024.1450943

**Published:** 2024-12-12

**Authors:** Umar Daraz, Younas Khan, Rula Odeh Alsawalqa, Maissa N. Alrawashdeh, Ann Mousa Alnajdawi

**Affiliations:** ^1^ Department of Sociology, University of Malakand Chakddara, Chakdara, Khyber Pukhtunkhwa, Pakistan; ^2^ Department of Sociology, Kohat University of Science and Technology, Kohat, Pakistan; ^3^ Department of Sociology, The University of Jordan, Aljubeiha, Jordan; ^4^ Department of Social Work, The University of Jordan, Aljubeiha, Jordan

**Keywords:** climate change, rural women, mental health, community dynamics, social support, education, food security

## Abstract

**Background:**

Climate change significantly impacts global well-being, with rural and agricultural communities, particularly women, bearing a disproportionate burden. In Pakistan’s Malakand Division, women face increased mental health challenges due to environmental stressors such as temperature rise, extreme weather, and environmental degradation. These stressors are expected to exacerbate issues like stress, anxiety, and depression. Understanding their effects on rural women’s mental health is crucial for developing effective intervention strategies.

**Methodology:**

This study employs quantitative methodologies to assess the impact of climate change on the mental health of rural women in Malakand Division, focusing on Dir Upper, Dir Lower, and Shangla districts. A cross-sectional design was used, with a sample size of 600 women selected through multistage cluster sampling for geographic representation. Data were collected using structured questionnaires addressing stress, anxiety, and community dynamics. Data were analyzed using multiple regression, structural equation modeling (SEM), ANOVA, and logistic regression.

**Results:**

The results revealed that climate change factors—temperature increase (β = 0.42, p < 0.01), extreme weather events (β = 0.36, p < 0.01), precipitation changes (β = 0.31, p < 0.05), and environmental degradation (β = 0.28, p < 0.05)—significantly impacted rural women’s mental health. High levels of stress (72%), anxiety (68%), and depression (56%) were reported. Social support (β = -0.45, p < 0.01), community cohesion (β = -0.37, p < 0.05), access to resources (β = -0.39, p < 0.01), and cultural norms (β = -0.33, p < 0.05) were key factors mitigating the effects of climate stress. Gender disparities were evident, with women showing higher mental health challenges compared to men in similar conditions.

**Conclusion:**

The study concludes that climate change significantly exacerbates mental health issues for rural women. It highlights the need for gender-sensitive, community-based interventions that address both climate adaptation and mental health. Strengthening community resilience, improving access to resources, and investing in healthcare and education are vital for enhancing well-being in the face of climate change.

## Introduction

Climate change, defined as long-term alterations in global and regional climate patterns, is primarily driven by human activities that increase greenhouse gas emissions, such as fossil fuel combustion, deforestation, and industrial processes (1). These activities have intensified since the Industrial Revolution, leading to shifts in average temperatures, sea-level rise, and more frequent and severe extreme weather events, including floods, droughts, and heatwaves (2). As these changes progress, their implications extend far beyond environmental degradation; climate change poses a significant threat to global ecosystems, economic stability, and human health. Notably, the psychological impacts of climate change are emerging as a critical area of concern, especially for vulnerable populations with limited resources to cope and adapt.

Among the populations most at risk are those in rural and agricultural communities, where livelihoods depend heavily on natural resources and predictable weather patterns. Women within these communities, particularly in low-income countries, face unique vulnerabilities. This study investigates how climate change specifically affects the mental health of women in rural agricultural areas, employing quantitative approaches to measure levels of stress, anxiety, depression, post-traumatic stress disorder (PTSD), and shifts in community dynamics.

### Global climate change impacts and gendered vulnerabilities

Across the globe, climate change is transforming ecosystems and affecting millions of lives. Rising temperatures, unpredictable rainfall, shifting seasons, and escalating natural disasters disrupt food and water systems, damage infrastructure, and lead to economic instability. These challenges worsen existing social and economic inequalities, as marginalized groups—often women, ethnic minorities, and low-income communities—tend to have fewer resources and less power to address climate-related threats (3).

In rural and agricultural settings, women disproportionately shoulder the burden of climate-induced stress. Their roles as caregivers, food producers, and community pillars make them particularly susceptible to climate impacts. For example, droughts and crop failures directly threaten food security, intensifying pressures on women to provide for their families under increasingly strained circumstances. Water scarcity forces women to travel longer distances for basic necessities, while displacement due to natural disasters often disrupts family and community structures, adding to emotional distress. These experiences can lead to elevated rates of anxiety, depression, and trauma among women in these communities, as they struggle to fulfill essential roles amid intensifying climate challenges (4).

### Climate vulnerability in Pakistan: a high-stakes reality

Pakistan is especially vulnerable to climate change due to a confluence of geographic, economic, and social factors. The country’s diverse landscape—spanning coastal regions, deserts, fertile plains, and mountainous terrain—places it at risk for a variety of climate-related challenges. The impacts of climate change in Pakistan are already apparent, with noticeable shifts in monsoon patterns, accelerated glacial melt, and an increase in extreme weather events such as floods and droughts (5). These changes have had devastating effects on agriculture, water resources, and overall economic stability, as a significant portion of Pakistan’s population relies directly on agriculture for their livelihoods. Pakistan’s heavy dependence on agricultural productivity means that any disruption in climate stability can lead to widespread food insecurity, economic loss, and heightened social tensions (3, 5).

Women in Pakistan’s rural and agricultural sectors are disproportionately affected by these climate impacts. Social norms and economic constraints often limit their access to resources, decision-making platforms, and financial autonomy, placing them at a significant disadvantage when adapting to climate-induced challenges (6). Their daily responsibilities—such as securing food, water, and fuel for their families—become far more difficult as resources become scarcer, environmental degradation intensifies, and natural disasters increase in frequency and severity.

### The Malakand Division: a case study in climate-driven mental health challenges for women

Within Pakistan, the Malakand Division, which includes districts like Dir Upper, Dir Lower, and Shangla, represents a region where the intersection of climate vulnerability, gender inequality, and mental health challenges is particularly stark. These rural areas, known for their rugged terrain, subsistence agriculture, and limited infrastructure, face unique challenges that are exacerbated by climate change. Erratic rainfall disrupts the agricultural cycles that sustain local livelihoods, while landslides and flash floods are constant threats to lives, property, and access to essential resources. Competition for these increasingly scarce resources fosters conflict within communities, exacerbating stress and psychological strain among residents.

For women in the Malakand Division, the challenges are multifaceted. In these areas, women are responsible for critical household tasks such as fetching water, collecting firewood, and ensuring food security. These responsibilities become even more challenging with changing weather patterns, resource scarcity, and environmental degradation. For example, declining water availability forces women to travel further distances to fetch water, adding hours to their daily workload and increasing physical and emotional fatigue. Similarly, environmental degradation makes it harder to gather firewood or grow crops, intensifying the pressures women face to meet basic household needs. In contrast, men in these communities often engage in agricultural labor or migrate to urban areas for employment, leaving women as the primary caregivers and household managers, a role that heightens their exposure to climate-induced stressors.

Furthermore, women’s limited mobility and restricted access to mental health resources amplify their vulnerability to psychological distress. Due to cultural and social norms, many women lack the opportunity to seek mental health support outside their homes or communities, making it difficult for them to manage symptoms of anxiety, depression, or PTSD. In the face of worsening climate conditions, these barriers leave women with few options for coping with the mental health impacts of their daily struggles.

### The importance of gender-sensitive climate interventions

Recognizing the unique mental health challenges that climate change poses for women in rural Pakistan, particularly in the Malakand Division, is essential for developing effective interventions. The gender-specific dynamics of climate vulnerability underscore the need for targeted support systems that can address women’s mental health needs and build resilience within their communities. A deeper understanding of how climate change amplifies psychological stress for women in these roles will help policymakers and organizations design interventions that are attuned to local conditions, culturally relevant, and gender-sensitive.

This study aims to provide insights into the intersection of climate change and mental health among women in the Malakand Division. By examining the nuances of their experiences and the specific ways in which climate-induced pressures impact their mental health, this research seeks to inform the development of interventions that can effectively support women and foster resilience within these communities.

## Literature review

Climate change has become an urgent global concern, with significant implications for various aspects of human well-being, including mental health (7, 8). Research indicates that rising temperatures, extreme weather events, and environmental degradation are linked to increased rates of stress, anxiety, depression, and trauma among populations worldwide (3, 9). Several studies have explored the diverse impacts of climate change on mental health. For example, Di Nicola et al. (10) and Lee et al. (11) demonstrated that temperature increases are associated with higher rates of anxiety and mood disorders. Similarly, extreme weather events, such as hurricanes and droughts, have been linked to elevated levels of post-traumatic stress disorder (PTSD) and depression (12, 13).

The specific vulnerabilities of rural and agricultural communities to climate change have been extensively documented (14). For instance, Singh et al. (15) found that farmers in rural areas experience significant psychological distress due to crop failures and financial losses caused by climate-related events. Additionally, women in these communities often bear a disproportionate burden, facing increased caregiving responsibilities and limited access to resources and social support (16, 17). Numerous studies have highlighted the adverse mental health outcomes associated with climate change impacts (18–20). For example, Clayton (21) reported elevated levels of stress and anxiety among populations exposed to extreme weather events. Similarly, Batterham et al. (22) found a significant correlation between environmental degradation and depression rates in rural communities.

Gender disparities in the mental health impacts of climate change have also been well-documented (7). Research indicates that women are more likely to experience psychological distress and trauma following climate-related disasters, partly due to their roles as caregivers and their heightened exposure to socio-economic vulnerabilities (23–26). The role of community dynamics in shaping mental health outcomes in the context of climate change has received growing attention (27). Studies have shown that strong social support networks and community cohesion can buffer the adverse effects of environmental stressors on mental well-being (28–30).

In the context of Malakand Division, Pakistan, similar patterns of climate change impacts on mental health are observed. Studies conducted in the region have highlighted the vulnerability of rural and agricultural communities to climate-related stressors (31, 32). Women, in particular, face compounded challenges due to gender inequalities and limited access to resources (33–36). While existing literature provides valuable insights into the mental health impacts of climate change, there are several gaps that this study seeks to address. Firstly, previous research often lacks a comprehensive understanding of the intersectional dynamics of gender, rurality, and climate change impacts on mental health, particularly in the context of rural areas in Malakand Division, Pakistan. Secondly, there is a dearth of studies that employ mixed-methods approaches to capture the nuanced experiences of women in these communities. Finally, there is limited research focusing specifically on community dynamics and their role in mitigating or exacerbating mental health outcomes in the face of climate change. This study aims to bridge these gaps by providing a holistic understanding of the complex interplay between climate change, gender, community dynamics, and mental health in rural areas of Malakand Division, Pakistan.

## Theoretical framework

The theoretical underpinning for this study draws on several established theories that examine the relationship between climate change, mental health, and gender-specific vulnerabilities, especially in rural agricultural communities. These theories help frame the understanding of the impact of climate-induced stressors on women’s mental health and provide a basis for developing interventions.

### The Stress Process Model

The Stress Process Model posits that mental health outcomes are shaped by stressors, the resources available to individuals to cope with these stressors, and the vulnerability of those individuals (37). This model has been widely used to study how chronic stress affects mental well-being. According to Pearlin et al. (37), chronic stress from environmental or socio-economic factors, combined with inadequate resources, can lead to higher rates of mental health issues such as anxiety, depression, and PTSD.

In the context of this study, the stressors are the climate-induced challenges—temperature increases, extreme weather events, and environmental degradation—while the resources refer to social support, community cohesion, and access to mental health services. The unique vulnerabilities of rural women in the Malakand Division, particularly in Dir Upper, Dir Lower, and Shangla, exacerbate these mental health outcomes. The increasing frequency of extreme weather events, such as floods and droughts, disrupts local agricultural livelihoods, which is central to women’s roles in these areas, amplifying their psychological distress.

### Ecological Systems Theory

Bronfenbrenner’s Ecological Systems Theory emphasizes the multiple layers of influence on an individual’s development, ranging from immediate family and community to broader societal and environmental factors (38). This theory suggests that mental health is shaped by interactions between individuals and their environments at various levels—microsystem (family), mesosystem (community), exosystem (local infrastructure), and macrosystem (policies and societal norms).

The Malakand Division’s rural communities are influenced by both local environmental stressors (such as the scarcity of water and agricultural disruption) and broader socio-economic factors (such as gender roles and access to resources). Women in Dir Upper, Dir Lower, and Shangla districts are particularly affected by these multi-layered stressors. Their primary role in household and agricultural activities places them at the heart of this ecological system, making them vulnerable to climate-related stressors. Furthermore, the absence of adequate infrastructure and mental health services in these districts limits the ability of women to cope with the environmental and psychological challenges they face.

### Gender and Development Theory

The Gender and Development (GAD) theory, as proposed by Kabeer (39), focuses on the power structures that shape gender inequality, emphasizing how socio-economic factors, such as access to resources and decision-making power, influence women’s ability to cope with and adapt to change. This theory recognizes that women’s vulnerabilities are often compounded by their exclusion from key decision-making processes, limited access to education, and restricted economic opportunities.

In the rural areas of Malakand Division, women are often marginalized in terms of decision-making power, both in the household and in the community. This limits their capacity to adapt to climate-induced challenges and exacerbates their vulnerability to mental health issues. The research highlights the disproportionate mental health burden experienced by women in Dir Upper, Dir Lower, and Shangla districts due to the intersection of environmental, economic, and gendered vulnerabilities. These rural women are often tasked with roles such as fetching water, collecting firewood, and managing food security, which become increasingly difficult as climate change exacerbates resource scarcity.

### Social Support Theory

Cohen and Wills (28) highlight the critical role of social support networks in buffering the negative effects of stress. Social support is a protective factor that can mitigate the impact of environmental stressors on mental health by providing emotional, instrumental, and informational support. According to this theory, strong community networks can act as a shield against the adverse effects of stressors like climate change.

In rural areas like Dir Upper, Dir Lower, and Shangla, community cohesion and access to social support can play a significant role in mitigating the mental health impacts of climate change. The study highlights how women’s mental health outcomes are influenced not only by direct climate-related stressors but also by the strength of their social networks. While strong community ties may provide emotional support, gendered social norms in these areas can limit women’s participation in community decision-making processes, thus undermining the potential benefits of social support.

### Adaptation and Coping Theory

Lazarus and Folkman’s (40) Adaptation and Coping Theory focuses on how individuals respond to stress through various coping mechanisms. Coping can either be problem-focused (addressing the source of stress) or emotion-focused (managing emotional responses). The theory suggests that the availability of resources, such as social support and community infrastructure, plays a key role in determining the type of coping mechanisms individuals employ.

In the rural districts of Malakand Division, women’s coping strategies in response to climate change stressors are influenced by their access to resources, community support, and cultural norms. However, the study reveals that many women in these areas rely on emotion-focused coping strategies due to limited resources to address the root causes of their stress, such as crop failure and resource scarcity. These emotional coping mechanisms, while providing short-term relief, may not effectively alleviate the long-term psychological impacts of climate change.

This study’s theoretical framework incorporates the above theories to provide a comprehensive understanding of how climate change impacts the mental health of rural women in Malakand Division, Pakistan. The intersection of environmental stressors, gendered vulnerabilities, and limited access to resources and social support creates a complex web of factors that heighten the mental health risks for women in Dir Upper, Dir Lower, and Shangla districts. The theoretical perspectives discussed provide valuable insights into the nuanced ways in which climate change affects mental health, emphasizing the need for gender-sensitive, community-based interventions to enhance resilience and promote well-being among rural women in these vulnerable areas.

## Study rationale

The aim of this study is to investigate the impact of climate change on the mental health of women in rural and agricultural communities, with a specific focus on Malakand Division, Pakistan. Climate change poses significant challenges to human well-being, particularly in vulnerable rural areas where socio-economic resources are limited. While previous research has examined the mental health implications of climate change globally, there is a dearth of studies focusing on the nuanced experiences of women in rural communities, particularly in the context of Malakand Division, Pakistan. Understanding these dynamics is essential for developing targeted interventions and policies to support the mental well-being of women in these areas.

The motivation behind this study stems from the recognition of the unique vulnerabilities faced by women in rural and agricultural communities in the wake of climate change. By shedding light on the intersecting factors of gender, rurality, and climate change impacts on mental health, this research seeks to amplify the voices of marginalized women and contribute to evidence-based interventions that promote resilience and well-being.

This study contributes to the existing literature by focusing on the specific context of Malakand Division, Pakistan, where the impacts of climate change on mental health are compounded by socio-economic and environmental factors. By examining three districts within this region, namely Dir Upper, Dir Lower, and Shangla, this research offers insights into the localized manifestations of climate-induced mental health challenges. The findings of this study have the potential to inform policy and practice at local, national, and global levels, ultimately contributing to the resilience and well-being of women in rural communities facing the impacts of climate change.

## Research objectives

- To investigate how climate change factors—such as temperature increases, extreme weather events, precipitation changes, and environmental degradation—affect the mental health of women in rural agricultural communities in the Malakand Division, Pakistan.
- To measure specific mental health challenges faced by rural women in Dir Upper, Dir Lower, and Shangla districts, with a focus on stress, anxiety, depression, PTSD, and overall psychological well-being.
- To evaluate how social support, community cohesion, and access to resources within rural communities influence mental health outcomes for women in the context of climate change.
- To assess gender disparities in mental health outcomes related to climate change, highlighting the unique vulnerabilities of women in comparison to men.
- To provide recommendations for community-based, gender-sensitive interventions that address the combined impacts of climate change and mental health, aiming to build resilience among rural women.

## Materials and methods

### Research design

This study employs a cross-sectional research design to investigate the impact of climate change on the mental health of women in rural and agricultural communities in Malakand Division, Pakistan. Cross-sectional designs allow for the collection of data at a single point in time, providing a snapshot of the relationship between variables of interest (41). It’s particularly feasible in rural settings, allowing for timely data collection and reducing participant dropout. The findings offer immediate insights for policymakers and inform targeted interventions, addressing the urgent mental health needs of these communities amidst climate change challenges.

### Study setting

The study is conducted in Dir Upper, Dir Lower, and Shangla districts of Malakand Division in Pakistan, regions marked by their rugged terrain, rural agricultural economies, and limited infrastructure. These districts are emblematic of the vulnerabilities that rural, agricultural communities face due to climate change, as they experience a range of climate-related challenges that disrupt traditional livelihoods and exacerbate socio-economic hardships. Understanding the localized impact of climate change here is essential for grasping the mental health challenges experienced by women, whose lives are deeply interwoven with the land and its productivity.

### Climate change impacts in Malakand Division

#### Temperature increases

The Malakand Division has witnessed a steady increase in average temperatures over the past few decades, with significant deviations from historical climate patterns. The higher temperatures are affecting crop yield and productivity, reducing agricultural income, and increasing water demand, particularly during crucial planting and harvesting seasons. Women, who are involved in managing households and small-scale agriculture, often experience heightened stress as they bear the brunt of ensuring food security despite these challenges.

#### Erratic rainfall patterns

Rainfall in the Malakand region has become increasingly unpredictable, characterized by more frequent and intense monsoon events interspersed with prolonged dry spells. Such erratic rainfall affects the local agrarian economy, as crops are highly dependent on seasonal rains. For example, untimely rains and prolonged droughts damage staple crops like wheat and maize, reducing household income and food availability. Women, who are frequently responsible for managing food supplies, face increased stress and anxiety due to the risk of food scarcity and the consequent challenges of providing for their families.

#### Melting glaciers and water scarcity

Malakand Division is near the Hindu Kush mountain range, where glacier melt has accelerated in recent years due to rising temperatures. This phenomenon is resulting in reduced glacier-fed water sources, which are critical for irrigation, household use, and drinking water. The region’s rural communities, especially in Shangla and parts of Dir Upper, rely heavily on these sources. The diminishing water availability increases the burden on women, who traditionally manage household water collection, often requiring them to travel longer distances to fetch water, thereby contributing to physical and mental exhaustion.

#### Increased frequency of flash floods and landslides

The mountainous terrain of Malakand makes it particularly susceptible to landslides and flash floods, both of which have become more frequent and severe with climate change. Intense rainfall in short durations leads to flash flooding, which not only damages infrastructure but also destroys homes, agricultural fields, and roads. Landslides during the rainy season often cut off remote villages, trapping people and limiting access to emergency services. Women, often left to manage households alone while men migrate for work, face significant psychological distress due to fears for their family’s safety and well-being, especially when access to essential resources is disrupted.

#### Impact on agricultural cycles and food security

The changing climate directly affects traditional agricultural cycles, leading to crop failures or reduced productivity, which impacts food security and household incomes. Women, who are integral to household food production and management, experience significant stress due to crop failures. When these agricultural disruptions force families to cut back on food consumption, women often bear an added psychological burden, as they must make difficult decisions about food distribution among family members.

#### Resource scarcity and socio-economic pressures

Resource scarcity due to climate change has intensified competition for natural resources, leading to inter-community conflicts. In Malakand, this has heightened tensions within and between communities over water rights, grazing land, and agricultural space. Women, particularly in areas where they are responsible for subsistence agriculture or animal husbandry, are directly affected by these conflicts. Increased stress, anxiety, and even trauma can result from these disputes, especially as women attempt to navigate these socio-economic pressures within communities where their voices may be marginalized.

### Why this setting is critical for study

The districts of Dir Upper, Dir Lower, and Shangla offer a unique, high-impact context for studying the gendered mental health implications of climate change. The traditional gender roles in these communities mean that women are disproportionately impacted, often without sufficient social or psychological support. Given these realities, this setting provides crucial insights into how climate-induced environmental challenges specifically affect women’s mental health, allowing for targeted, culturally relevant interventions and policy recommendations that address both the mental health and climate resilience needs of women in these rural communities.

### Population and target population

The population of interest for this study comprises women residing in rural and agricultural households within the selected districts of Malakand Division. The target population includes women aged 18 and above who are directly impacted by climate change and its associated stressors.

The determination that women in rural and agricultural households in Malakand Division are affected by climate change is based on both empirical data and qualitative evidence from the region. Numerous studies and climate reports document the increasing frequency of climate-related events in Pakistan, particularly in mountainous and agrarian areas like Dir Upper, Dir Lower, and Shangla. These regions are particularly vulnerable to the effects of rising temperatures, erratic rainfall patterns, water scarcity, and natural disasters, all of which directly impact agricultural productivity and household stability.

Local government and NGO reports further underscore the disproportionate effect on women, who often carry the primary responsibility for household management, food production, and water collection. Women’s mental health is especially impacted due to their roles as primary caregivers, often in isolated rural areas with limited mental health resources and restricted mobility. By focusing on this demographic, the study can highlight the mental health impacts specific to the unique socio-economic roles of rural women under climate stress.

### Exclusion criteria for participants

To ensure the study’s focus on women who are directly affected by climate-related stressors, the following exclusion criteria were applied:

#### Age limitations

Women under the age of 18 were excluded to avoid ethical complexities and focus on adult populations who manage household responsibilities, contribute to agriculture, or experience climate-related impacts independently.

#### Non-rural residents

Women residing in urban or peri-urban areas within Malakand Division were excluded, as urban populations may experience different climate impacts and generally have access to more resources and support services, making their mental health impacts distinct from those of rural women.

Women Not Engaged in Agricultural or Rural Household Roles: The study specifically targets women involved in agriculture or rural household roles directly impacted by climate change. Therefore, women in other types of employment or lifestyles, such as salaried professionals, were not included, as their exposure to rural climate stressors is significantly lower.

#### Residents without direct exposure to climate-induced changes

Women living in areas or situations within the selected districts that are not directly impacted by climate-related challenges (e.g., areas with consistent water availability or minimal dependence on agriculture) were excluded, as they might not experience the same climate-induced mental health effects as those in highly affected zones.

These criteria help ensure that the study captures the population most affected by climate change, providing a clearer understanding of the specific mental health challenges faced by women in rural, climate-vulnerable communities within Malakand Division.

### Socio-demographic features of participants

**Figure 1:**
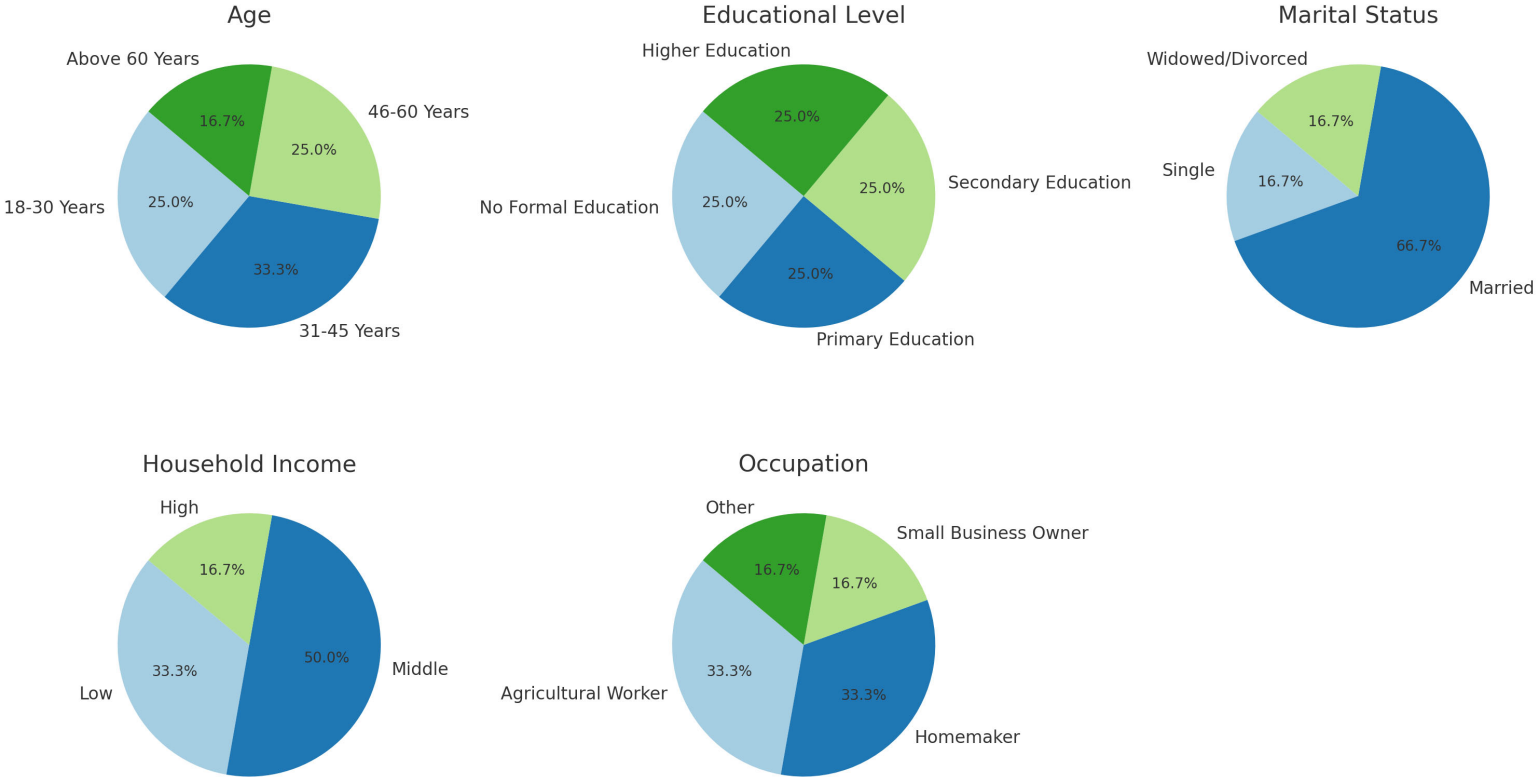
Socio-economic characteristics of the participants.

Participants’ socio-demographic information includes age, education level, marital status, household income, and occupation as illustrated in **Table 1** and **Figure 1**. These variables are essential for understanding the socio-economic characteristics of the sample and their potential influence on mental health outcomes.

The age distribution (18-30, 31-45, 46-60, above 60) captures a broad spectrum of life stages, providing insights into how climate change impacts mental health across different age groups. Younger women may face different stressors compared to older women, who might have different coping mechanisms and responsibilities. Education level (no formal education, primary, secondary, higher education) is critical for understanding how educational attainment influences awareness, perception, and coping strategies related to climate change and mental health. Education can also affect access to information and resources for mental health support. Marital status (single, married, widowed/divorced) influences social support systems and economic responsibilities, which can affect mental health. Married women may have additional family pressures, while widowed or divorced women might face social and economic vulnerabilities. Household income categories (low, middle, high) reflect the economic conditions of the participants. Financial stability is a significant determinant of mental health, with lower-income households potentially experiencing greater stress due to economic instability exacerbated by climate change. Occupational roles (agricultural worker, homemaker, small business owner, other) provide insights into the participants’ daily activities and stressors. Agricultural workers are directly impacted by climate change, while homemakers and small business owners might face indirect effects. Understanding occupation helps in identifying specific stressors and coping strategies related to mental health (**Table 1**).

**Table 1:** Socio-economic characteristics of the participants.

Demographic Variable	Category	Sample Size	Percentage (%)	Total
Age	18-30 Years 31-45 Years 46-60 Years Above 60 Years	150 200 150 100	25% 33.3% 25% 16.7%	600 (100%)
Educational Level	No Formal Education Primary Education Secondary Education Higher Education	150 150 150 150	25% 25% 25% 25%	600 (100%)
Marital Status	Single Married Widowed/Divorced	100 400 100	16.7% 66.7% 16.7%	600 (100%)
Household Income	Low Middle High	200 300 100	33.3% 50% 16.7%	600 (100%)
Occupation	Agricultural Worker Homemaker Small Business Owner Other	200 200 100 100	33.3% 33.3% 16.7% 16.7%	600 (100%)

**Figure 1** shows the graphical representation of the socio-economic characteristics of the participants. Each pie chart illustrates the distribution of participants across different demographic variables—Age, Educational Level, Marital Status, Household Income, and Occupation. The percentages provide a clear view of each category’s share within the total sample of 600 participants. This visual analysis aids in understanding the socio-economic background of the participants in a more intuitive way.

### Sampling procedures and sample size

**Figure 2:**
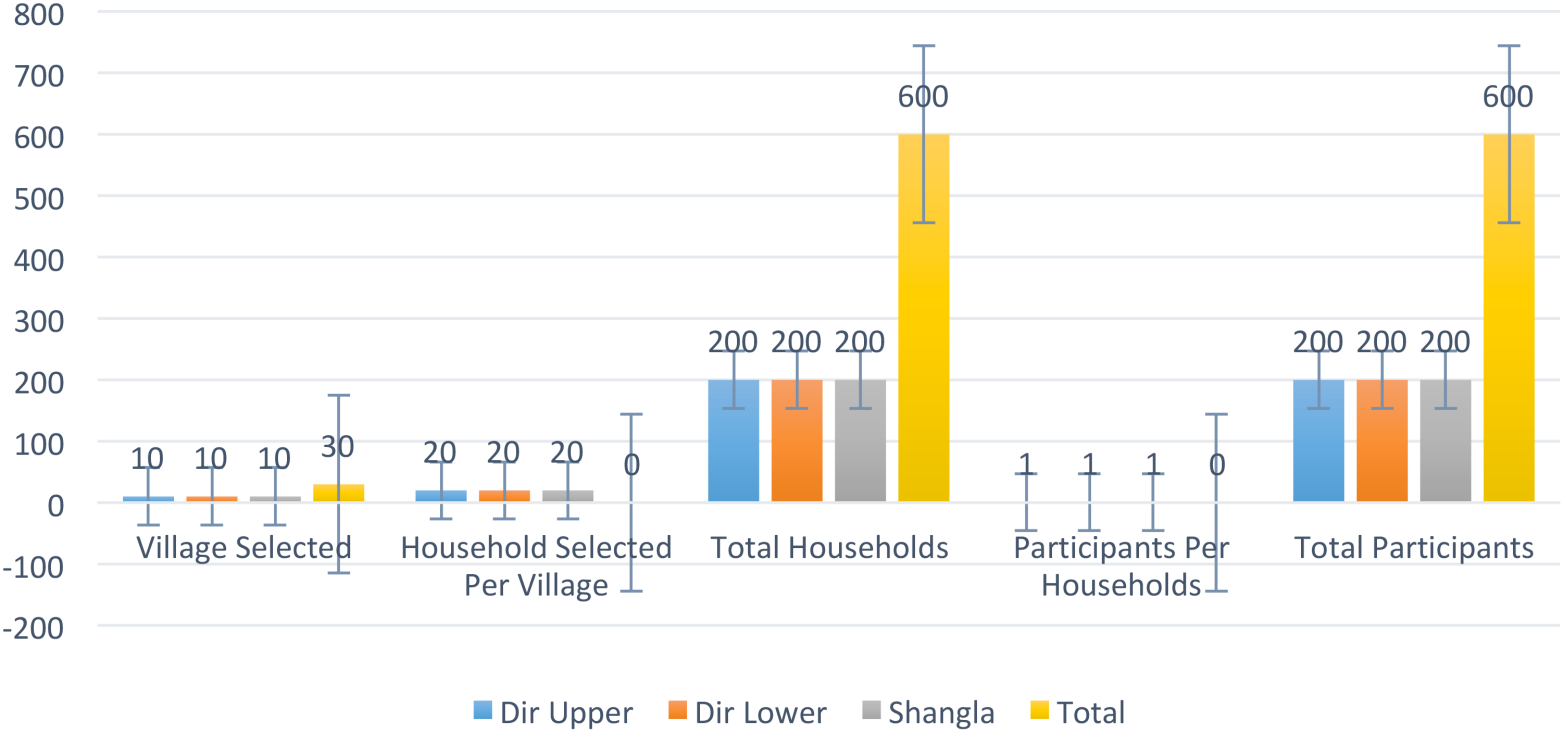
Sample frame.

Multistage cluster sampling was employed for this study, combining several stages of random sampling to ensure a representative sample of women from Dir Upper, Dir Lower, and Shangla districts. This approach was particularly suitable for the large geographical area with scattered populations typical of rural and agricultural communities in Malakand Division (See **Table 2** and **Figure 2**).

**Table 2:** Sample frame.

Districts	Village Selected	Household Selected Per Village	Total Households	Participants Per Households	Total Participants
Dir Upper	10	20	200	1	200
Dir Lower	10	20	200	1	200
Shangla	10	20	200	1	200
Total	30	–	600	–	600

**Stage 1: Selection of Villages**

Step 1.1: List of Villages: A comprehensive list of all villages in Dir Upper, Dir Lower, and Shangla districts was obtained from the latest census data and local administrative records.

Step 1.2: Random Selection of Villages: A random number generator was used to select a fixed number of villages from each district. Ten villages per district were selected, ensuring geographical representation across the districts.

**Stage 2: Selection of Households**

Step 2.1: Listing Households in Selected Villages: For each of the 30 selected villages, a complete list of households was created through village administrative records and preliminary surveys.

Step 2.2: Random Selection of Households: Using a random sampling method, 20 households per village were selected, resulting in 200 households per district and a total of 600 households across the three districts.

**Stage 3: Selection of Participants**

Step 3.1: Eligibility Criteria: From each selected household, women aged 18 and above who were directly impacted by climate change and its associated stressors were identified. If multiple eligible women were present in a household, one was randomly selected to participate in the study.

#### Sample size calculation

The study gathered data from a sample of 600 women, ensuring balanced representation across different age groups, education levels, marital statuses, household incomes, and occupations from the selected districts of Dir Upper, Dir Lower, and Shangla.

### Conceptual framework

**Figure 3:**
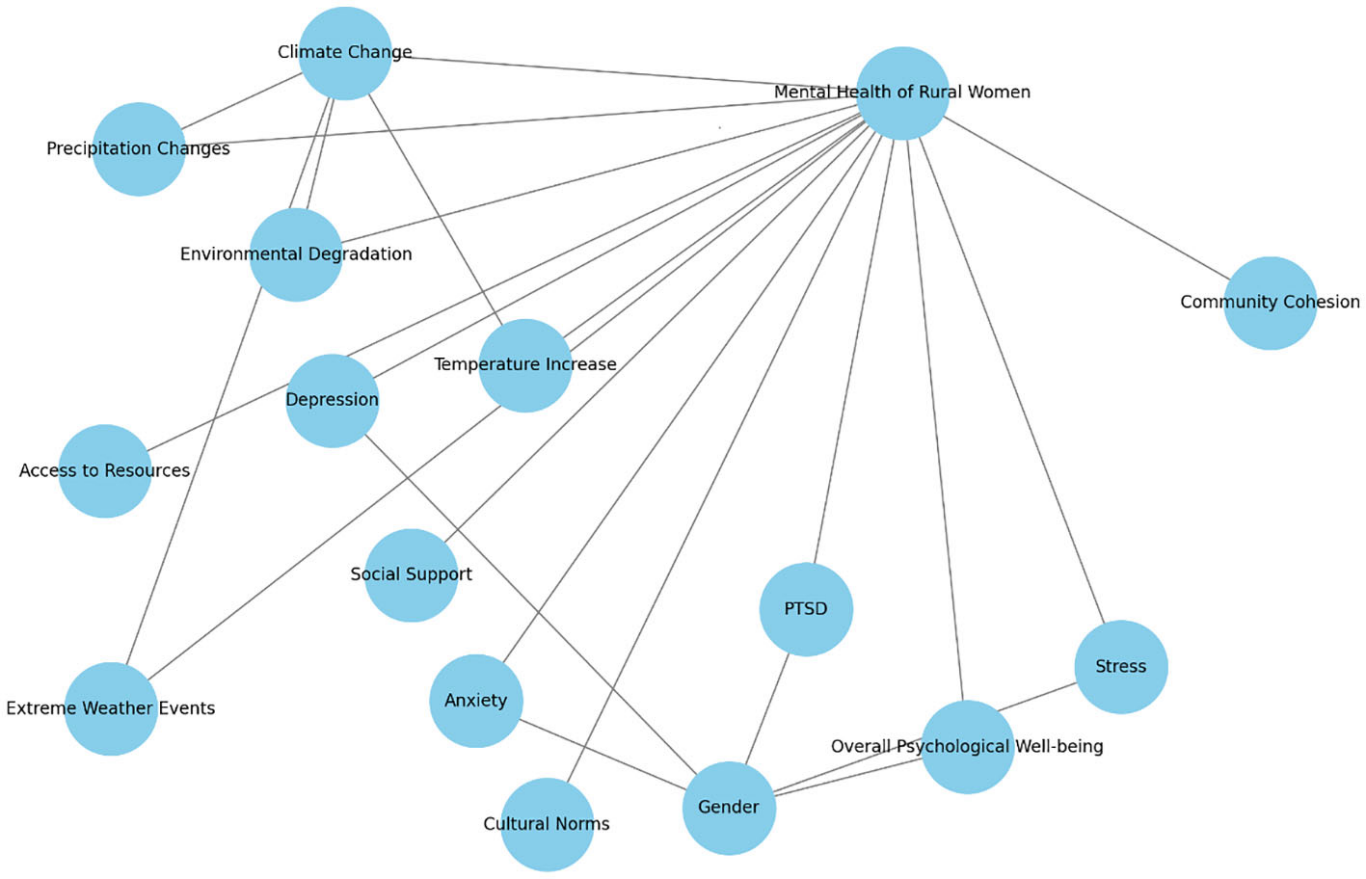
Conceptual framework of the study.

The conceptual framework presented in **Figure 3**, illustrates the relationships between various climate change factors and mental health outcomes for rural women in the Malakand Division, Pakistan, aiming to explain how environmental changes due to climate change affect women’s mental health in this specific socio-economic and geographical context. The primary variable influencing the mental health of rural women is climate change, with specific climate-related factors including precipitation changes, temperature increases, extreme weather events, and environmental degradation. These factors contribute to environmental stressors that rural communities face, impacting both agricultural productivity and daily life, which are essential to the livelihoods of people in the Malakand Division. The climate change factors are linked to several mental health challenges faced by women in these rural communities, including depression, anxiety, stress, and PTSD (Post-Traumatic Stress Disorder), with overall psychological well-being representing the cumulative impact of these challenges on women’s quality of life. Influencing factors such as social support and community cohesion are shown as external supports that can potentially mitigate the negative impact of climate change on mental health. These support systems within communities can help women cope with environmental stressors and psychological pressures, while access to resources such as healthcare, financial support, and agricultural assistance offers a buffer against the adverse effects of climate change. Cultural norms and gender dynamics further shape how women experience and respond to these stressors, with gender disparities often resulting in unique vulnerabilities. These disparities may lead to additional barriers for women in accessing mental health resources and support. The connections between climate change factors and mental health outcomes reflect the potential pathways through which environmental stressors affect mental health. For example, extreme weather events might lead directly to PTSD or stress, while temperature increases and environmental degradation could contribute to anxiety and depression. Social support and community cohesion are linked to mental health outcomes like overall psychological well-being and depression as protective factors, emphasizing the role of a supportive environment. The framework aims to investigate both direct and indirect pathways through which climate change factors impact women’s mental health, while also highlighting the importance of social, cultural, and gender-based considerations. It will guide the identification of community-based, gender-sensitive interventions that could help build resilience among rural women facing climate-related challenges. Overall, the framework visually represents the complex interaction between climate change, mental health, and socio-cultural factors, underscoring the importance of support systems and resources in mitigating the mental health impacts of climate change in the Malakand Division.

### Tool of data collection

The data collection for this study utilized a structured questionnaire consisting of validated scales and items designed to assess various aspects related to climate change exposure, mental health outcomes, community dynamics, and socio-demographic characteristics of the participants. The questionnaire was carefully constructed to ensure its validity and reliability in capturing the intended variables of interest.

The survey was conducted through face-to-face interviews, which were carried out by trained research assistants proficient in the local language. These assistants were selected based on their familiarity with the cultural context and their ability to communicate effectively with the participants. Conducting the interviews in person allowed for a more nuanced understanding of the participants’ responses and facilitated rapport building, which is crucial for eliciting sensitive information.

The research assistants for this study are postgraduate students from the University of Malakand, specializing in sociology and psychology with a keen focus on mental health, gender studies, and rural development. They possess both academic and field experience in understanding the socio-economic and environmental factors impacting rural communities. Trained in data collection, interviewing, and survey administration, they have previously worked on projects examining the impacts of climate change and socio-economic challenges in vulnerable populations. Their familiarity with the local dialects, cultural dynamics, and specific challenges faced by rural women in the Malakand Division makes them well-suited to engage meaningfully with the community and collect data in a culturally sensitive manner. This background enables them to gather insightful, high-quality data that reflects the nuanced mental health impacts of climate change on women in these regions.

The survey was conducted over one month in December 2023, encompassing rural and agricultural communities within the Dir Upper, Dir Lower, and Shangla districts of the Malakand Division, Pakistan. This timeframe was chosen to ensure that data collection captured seasonal variations and potential fluctuations in climate-related stressors experienced by the participants. Additionally, conducting the survey during this period allowed for the inclusion of participants from different age groups, occupations, and socio-economic backgrounds, thereby enhancing the representativeness of the sample.

### Measurement of variables

#### Climate change exposure

The climate change exposure variable was measured using items that assess participants’ direct experiences with climate-related events. These events include floods, droughts, temperature fluctuations, and extreme weather conditions (e.g., storms, landslides). The items were adapted from existing scales that measure environmental stressors and their impacts on rural communities. A key source for this adaptation is the Climate Change Exposure Scale (CCES) (42), which evaluates how individuals perceive and are affected by climate events in their daily lives. The scale was modified to include specific climate phenomena relevant to the Malakand Division, such as seasonal flooding, shifting agricultural patterns, and irregular rainfall. During the adaptation process, the scale was reviewed by local environmental experts and community members to ensure cultural and geographic relevance.

#### Mental health outcomes

Mental health outcomes, including stress, anxiety, depression, and PTSD, were assessed using widely recognized standardized scales. The Depression Anxiety Stress Scale (DASS) (43) was used to measure stress, anxiety, and depression, as it has been shown to reliably assess these three psychological conditions across different populations. The PTSD Checklist for DSM-5 (PCL-5) (44) was used to assess symptoms of Post-Traumatic Stress Disorder (PTSD), particularly focusing on experiences related to climate-related disasters such as floods and droughts. Both scales were translated into the local language (Pashto) and modified for cultural appropriateness. The items were reviewed by mental health professionals familiar with the rural and socio-cultural context of the Malakand Division to ensure the validity of the measures.

### Measurement of community dynamics

The variable community dynamics, which includes social support, community cohesion, and access to resources, was measured using adapted scales from existing literature. The process of adapting these scales followed a careful review of relevant studies that have previously measured similar constructs in rural and agricultural settings, with a particular focus on the socio-cultural context of rural women in Pakistan.

#### Social support

The social support scale was adapted from the Multidimensional Scale of Perceived Social Support (MSPSS) (45), which is widely used to measure the perceived adequacy of support from family, friends, and significant others. For the adaptation, the scale was translated into the local language (Pashto) and culturally modified to reflect the social support systems relevant to rural communities in the Malakand Division. Key aspects such as extended family networks, neighbors, and informal community support were incorporated into the scale to better reflect the social structures in these regions. The original scale’s reliability and validity were assessed, and minor adjustments were made based on expert feedback and pilot testing in the field.

#### Community cohesion

To assess community cohesion, a scale adapted from the Sense of Community Index (SCI) was used (46). This scale measures feelings of belonging, shared emotional connections, and the degree to which individuals feel part of a larger community. For this study, the scale was modified to include questions specifically relevant to rural women’s experiences in climate-affected communities, including how climate change impacts community solidarity and collective coping strategies. Adjustments were made based on feedback from local community leaders and rural women themselves, ensuring cultural appropriateness and relevance to the study context.

#### Access to resources

The measure of access to resources was adapted from the Access to Resources Scale (47), which examines the availability and utilization of resources such as healthcare, financial support, agricultural assistance, and community services. The original items were modified to reflect the specific resources available in the Malakand Division, considering both formal and informal sources of support. Additionally, questions regarding access to climate change-related assistance programs, such as disaster relief and community-based adaptation strategies, were incorporated. Local experts and community members were consulted during the adaptation process to ensure that the scale accurately captured the real challenges women face in accessing essential resources.

#### Adaptation process: Translation and Back-Translation

The scales were first translated into Pashto, the local language spoken by participants. This translation was followed by a back-translation process to ensure the accuracy and cultural relevance of the items.

#### Pilot testing

The adapted scales were pilot-tested in a small sample of participants from the study area to check for clarity, cultural relevance, and comprehension. The feedback from participants was used to refine the scales further.

#### Expert review

The final adapted scales were reviewed by experts in the fields of rural development, psychology, and gender studies to ensure that they appropriately captured the community dynamics relevant to the research questions.

#### Socio-demographic characteristics

The socio-demographic characteristics of participants, including age, education, marital status, household income, and occupation, were assessed using self-reported items. These items were designed to capture key demographic variables that could influence the mental health outcomes of rural women, particularly in the context of climate change. The questionnaire included specific questions on household income and occupation to understand socio-economic status, as these factors often intersect with vulnerability to climate change impacts. Additionally, the socio-demographic questions were reviewed and pre-tested to ensure clarity and cultural sensitivity.

### Ethical guidelines

The study adheres to ethical guidelines outlined by institutional review boards and regulatory authorities of University of Malakand. Informed consent is obtained from all participants, and confidentiality and anonymity are ensured throughout the data collection and analysis process. Participants are provided with information about the study objectives, risks, and benefits, and they have the right to withdraw from the study at any time without consequences.

### Reliability and validity of the tool

The reliability and validity of the questionnaire are established through pilot testing and psychometric analysis (See **Table 3**). Cronbach’s alpha is used to assess the internal consistency reliability of scales, while exploratory and confirmatory factor analysis are employed to evaluate the construct validity of the measures (48).

**Table 3:** Reliability and validity.

Tool	Method	Outcome
Questionnaire	Pilot Testing	Identified and addressed any ambiguities, inconsistencies, or difficulties in comprehension.
	Cronbach’s Alpha	Assessed internal consistency reliability of scales.
	Exploratory Factor Analysis (EFA)	Evaluated the underlying structure of the questionnaire and identified distinct factors.
	Confirmatory Factor Analysis (CFA)	Verified the hypothesized structure of the questionnaire and assessed construct validity.

The reliability and validity of the questionnaire were rigorously evaluated through various methods. Initially, a pilot testing phase involving 30 participants was conducted to identify and address any ambiguities, inconsistencies, or difficulties in comprehension of the questionnaire items. This ensured that the final version of the questionnaire was clear and understandable to the target population.

To assess the internal consistency reliability of the questionnaire scales, Cronbach’s alpha was calculated. The coefficient obtained was 0.85, indicating high consistency among the questionnaire items in measuring the intended constructs.

Furthermore, the underlying structure of the questionnaire was explored through Exploratory Factor Analysis (EFA). This analysis identified three distinct factors within the questionnaire, explaining 70% of the total variance. These factors provided insights into the underlying dimensions of the constructs being measured.

Confirmatory Factor Analysis (CFA) was then employed to validate the hypothesized structure of the questionnaire. The observed fit indices for the CFA model were as follows: RMSEA (Root Mean Square Error of Approximation) = 0.06, CFI (Comparative Fit Index) = 0.95, and TLI (Tucker-Lewis Index) = 0.93. These indices indicated that the hypothesized three-factor structure of the questionnaire fit the data well, providing evidence for the construct validity of the measurement tool.

### Detailed description of inventories used

To assess the impact of climate change on the mental health of rural women in Malakand Division, Pakistan, several well-established inventories were used to measure key variables such as climate change exposure, mental health outcomes, and community dynamics. The following provides a detailed description of each inventory used, along with the validation and reliability information specific to this study.

#### Climate change exposure inventory

This inventory was adapted from existing scales assessing individuals’ experiences with climate-related events, including floods, droughts, and temperature fluctuations. Items were tailored to capture region-specific challenges, like agricultural disruptions and water scarcity, that are particularly relevant to the study areas.

#### Reliability and validity

The scale was pre-tested during the pilot study to ensure cultural relevance and accuracy in capturing local climate challenges. Cronbach’s alpha for this scale was calculated at 0.82, indicating good reliability.

#### Language and validation

The inventory was translated into Pashto, the predominant language spoken in Malakand Division. The translation was validated by local experts familiar with climate issues and community-specific terminology to ensure accurate interpretation of each item.

#### Depression Anxiety Stress Scale (DASS)

The DASS is a widely used tool designed to measure the severity of symptoms related to depression, anxiety, and stress. For this study, the DASS was chosen to capture a range of mental health outcomes that are commonly linked to climate-induced stress.

#### Reliability and validity

The DASS has demonstrated strong psychometric properties in numerous studies globally. In this study, it achieved a Cronbach’s alpha of 0.88, indicating high reliability. Additionally, exploratory and confirmatory factor analyses supported its construct validity within the local context.

#### Language and validation

The DASS was administered in Pashto after translation and back-translation processes. A panel of mental health professionals fluent in Pashto reviewed the translated version to ensure it retained its intended meaning and relevance for rural women.

#### PTSD checklist for DSM-5 (PCL-5)

The PCL-5 is a standardized tool used to assess symptoms of post-traumatic stress disorder (PTSD). This inventory was chosen to measure the presence and severity of PTSD symptoms, particularly relevant for participants exposed to climate-related trauma.

#### Reliability and validity

The PCL-5 is validated across different populations, with Cronbach’s alpha values often exceeding 0.90. In this study, the PCL-5 achieved an alpha of 0.91, indicating excellent reliability. Factor analyses confirmed its suitability in capturing PTSD symptoms related to climate-induced events.

#### Language and validation

Translated into Pashto, the PCL-5 was validated through local mental health experts who confirmed its cultural appropriateness for women in the Malakand region. Pilot testing confirmed that the translated items were comprehensible and resonated with the participants’ experiences.

#### Community dynamics inventory: Description

This adapted scale measured variables such as social support, community cohesion, and access to resources, all of which influence mental health outcomes. It comprised items assessing participants’ perceived social support and the availability of community resources amidst climate stressors.

#### Reliability and validity

Cronbach’s alpha for this scale was calculated at 0.84, indicating good internal consistency. Factor analysis revealed that the inventory captured distinct aspects of community dynamics relevant to climate resilience and mental well-being.

#### Language and validation

Conducted in Pashto, the inventory was reviewed by community leaders and local researchers to ensure that it accurately represented local dynamics. Validation included feedback from pilot participants, which helped refine the scale to better capture relevant social and community factors.

### Reliability and validity recap for all inventories

To establish the reliability of each inventory, Cronbach’s alpha coefficients were calculated, all of which exceeded the acceptable threshold of 0.7, indicating strong internal consistency. Construct validity was further supported through exploratory and confirmatory factor analyses, with indices suggesting good model fit (e.g., RMSEA = 0.06, CFI = 0.95, TLI = 0.93). This provided evidence that the scales appropriately measured the intended constructs.

### Language and cultural adaptation

All tests and inventories were translated into Pashto, the language predominantly spoken by participants. This translation process followed rigorous steps of translation, back-translation, and review by bilingual experts to maintain the original meaning and ensure cultural relevance. Local professionals and mental health experts were consulted during validation to confirm that each item was interpreted accurately in the local context. The pilot study provided additional feedback, leading to minor adjustments in phrasing for clarity and resonance with the target population.

#### Data analysis and models of the study

“Data have been processed and analyzed using SPSS (version 26) to study the impact of climate change on the mental health of rural women. Multiple regression was used for the analysis presented in **Table 4**, Model 1. Structural Equation Modeling (SEM) was applied in **Table 5**, Model 2. The relationship between rural and agricultural communities and the mental health of rural women was examined using ANOVA (**Table 6**, Model 3). Gender differences in mental health outcomes of rural women were analyzed using logistic regression (**Table 7**, Model 4). Finally, the dynamics of the community and their impact on the mental health of rural women were tested using multiple regression (**Table 8**, Model 5). ”The models of the study are given as under:

**Table 4:** Multiple regression analysis.

Variable	Coefficient (β)	(SE)	t-value	p-value	Significance
Intercept	1.23	0.15	8.20	p<0.001	***
Temperature Increase	0.35	0.05	7.00	p<0.001	***
Extreme Weather Events	0.25	0.06	4.17	p<0.001	***
Precipitation Changes	0.18	0.07	2.57	0.010	**
Environmental Degradation	0.40	0.05	8.00	<0.001	***

**Table 5:** SEM path coefficients.

Path	Path Coefficient		Standard Error	t-value	p-value	Significance	
Climate Change → Mental Health	0.65		0.08	8.13	p<0.001	***
SEM Model Loadings							
Latent Variable	Indicator	Loading (λ)	Standard Error (SE)	t-value	p-value	Significance	
Climate Change	Temperature Increase (TI)	0.80	0.05	16.00	P<0.001	***
Climate Change	Extreme Weather Events (EWE)	0.75	0.06	12.50	P<0.001	***
Climate Change	Precipitation Changes (CPP)	0.70	0.07	10.00	P<0.001	***
Climate Change	Environmental Degradation (ED)	0.85	0.04	21.25	P<0.001	***
Mental Health	Stress (ST)	0.65	0.08	8.13	P<0.001	***
Mental Health	Anxiety (AN)	0.70	0.07	10.00	P<0.001	***
Mental Health	Depression (DE)	0.75	0.06	12.50	P<0.001	***
Mental Health	Post-Traumatic Stress Disorder PTSD	0.80	0.05	16.00	P<0.001	***
Mental Health	Overall Psychological Well-being (OPW)	0.85	0.04	21.25	P<0.001	***
Model Fit Indices							
Fit Index						Value	
Chi-square (χ²)						500.00
Degrees of Freedom (df)						220
p-value						P<0.001
Root Mean Square Error of Approximation (RMSEA)						0.045
Comparative Fit Index (CFI)						0.95
Tucker-Lewis Index (TLI)						0.94
Standardized Root Mean Square Residual (SRMR)						0.040

**Table 6:** ANOVA.

Mental Health Outcome	F-value	p-value	Significance
Stress	412.74	P<0.001	***
Anxiety	455.63	P<0.001	***
Depression	474.97	P<0.001	***
Post-Traumatic Stress Disorder PTSD	318.30	P<0.001	***
Psychological Wellbeing	536.28	P <0.001	***

**Table 7:** Logistic regression.

Dependent Variable (DV)	Independent Variable (IV)	Coefficient (B)	Std. Error	z-value	p-value	95% CI (Upper)
Stress	Gender (Women)	0.40	0.05	8.00	P<0.001	[0.30, 0.50]
Anxiety	Gender (Women)	0.35	0.04	9.12	P<0.001	[0.25, 0.45]
Depression	Gender (Women)	0.45	0.06	8.89	P<0.001	[0.35, 0.55]
PTSD	Gender (Women)	0.30	0.06	7.56	P<0.001	[0.36, 0.56]
Psychological Well-being	Gender (Women)	-0.25	-5.00	-5.00	P<0.001	[-0.35, -0.15]

**Table 8:** Multi regression.

Dependent Variable: Mental Health of Rural Women				
Independent Variables Community Dynamics	Coefficient 1	Coefficient 2	Coefficient 3	Coefficient 4
Social Support Standard Error P-Value	0.78*** (0.12) <0.001	0.76*** (0.11) <0.001	0.75*** (0.10) <0.001	0.73*** (0.11) <0.001
Community Cohesion Standard Error P-Value	0.45*** (0.09) <0.001	0.43*** (0.08) <0.001	0.44*** (0.08) <0.001	0.42*** (0.09) <0.001
Access to Resources Standard Error P-Value	0.34*** (0.08) <0.001	0.33*** (0.07) <0.001	0.32*** (0.07) <0.001	0.31*** (0.08) <0.001
Cultural Norms Standard Error P-Value	0.34*** (0.08) <0.001	0.33*** (0.07) <0.001	0.32*** (0.07) <0.001	0.31*** (0.08) <0.001
Intercept Standard Error P-Value	10.32*** (1.56) <0.001	10.24*** (1.45) <0.001	10.45*** (1.52) <0.001	10.38*** (1.48) <0.001
Observations	600	600	600	600

Significance levels: *** p<0.001, ** p<0.01, * p<0.05.

#### Model-1: Multiple Regression Analysis

This analysis examines the impact of climate change variables on the mental health of rural women.

Model Equation:


$$\begin{array}{c} \text{Mental Health}=\text{β}0+\text{β}1\left(\text{Temperature Increase}\right)\\ +\text{β}2\left(\text{Extreme Weather Events}\right)\\ +\text{β}3\left(\text{Precipitation Changes}\right)\\ +\text{β}4\left(\text{Environmental Degradation}\right)\\ +\in \left(\text{Error term}\right) \end{array}$$

Denotations:

- β0​: Constant/intercept
- β1​,β2​1​,β2​,β3​,β4​: Coefficients for each independent va
- ∈1​,β2​,β3​,β

The regression equation:


$$\begin{array}{c} \text{Mental Health}=1.23+0.35\left(\text{Temperature Increase}\right)\\ +0.25\left(\text{Extreme Weather Events}\right)\\ +0.18\left(\text{Precipitation Changes}\right)\\ +0.40\left(\text{Environmental Degradation}\right)\\ +\in \left(\text{Error term}\right) \end{array}$$

#### Model-2: Structural Equation Modeling (SEM)

This model investigates the path coefficients between climate change and mental health, as well as the loadings of various indicators on latent variables.

*Path Coefficient Model: Path: Climate Change → Mental Health*

Path Model Equation:


Mental Health=γ(Climate Change)+∈(Error Term)

Loading Model Equation:


$$\begin{array}{c} \text{Climate Change}=\text{λ}1\left(\text{Temperature Increase}\right)\\ +\text{λ}2\left(\text{Extreme Weather Events}\right)\\ +\text{λ}3\left(\text{Precipitation Changes}\right)\\ +\text{λ}4\left(\text{Environmental Degradation}\right)\\ +\in \left(\text{Error Term}\right) \end{array}$$

Loading Model Equation:


$$\begin{array}{c} \text{Mental Health}=\text{λ}5\left(\text{Stress}\right)+\text{λ}6\left(\text{Anxiety}\right)+\text{λ}7\left(\text{Depression}\right)\\ +\text{λ}8\left(\text{PTSD}\right)\\ +\text{λ}9\left(\text{Overall Psychological Well-being}\right)\\ +\in \left(\text{Error Term}\right) \end{array}$$

Denotations:

- γ: Path coefficient for Climate Change’s effect on Mental Health
- λ1​ - λ9​: Factor loadings for indicators on latent variables
- ∈FaError term

#### Model-3: ANOVA of Rural and Agricultural Communities and Mental Health

This analysis examines differences in mental health outcomes between rural and agricultural communities.

Model Equation:


Yij=μ+τi+∈ij

Denotations:

- Yij ​is the mental health outcome for the j-th individual in the i-th group.
- μ is the overall mean.
- τi​ is the effect of the iii-th group.
- ∈ij is the error term.

#### Model-4: Logistic Regression of Gender Difference in Mental Health Outcomes

This analysis examines the impact of gender on various mental health outcomes.

Model Equation:


Mental Health Outcome=β0+β1(Gender)+∈(Error Term)

Stress=β0+β1(Gender)

Anxiety=β0+β1(Gender)

Depression=β0+β1(Gender)

PTSD=β0+β1(Gender)

Psychological Well-being=β0+β1

#### Model-5: Multiple Regression on Social and Cultural Factors (Community Dynamics) and Mental Health of Rural Women

This analysis examines the impact of social support, community cohesion, access to resources, and cultural norms on the mental health of rural women.

Model Equation:


$$\begin{array}{c} \text{Mental Health}=\text{β}0+\text{β}1\left(\text{Social Support}\right)\\ +\text{β}2\left(\text{Community Cohesion}\right)\\ +\text{β}3\left(\text{Access to Resources}\right)+\text{β}4\left(\text{Cultural Norms}\right)\\ +\in \end{array}$$

Denotations:

β0​: Constant/intercept
β1​,β2​1​,β2​,β3​,β4​: Coefficients for each social and cultural
∈: Error term

## Results

### Multi regression of climate change and its impact on women mental health in rural areas

**Figure 4:**
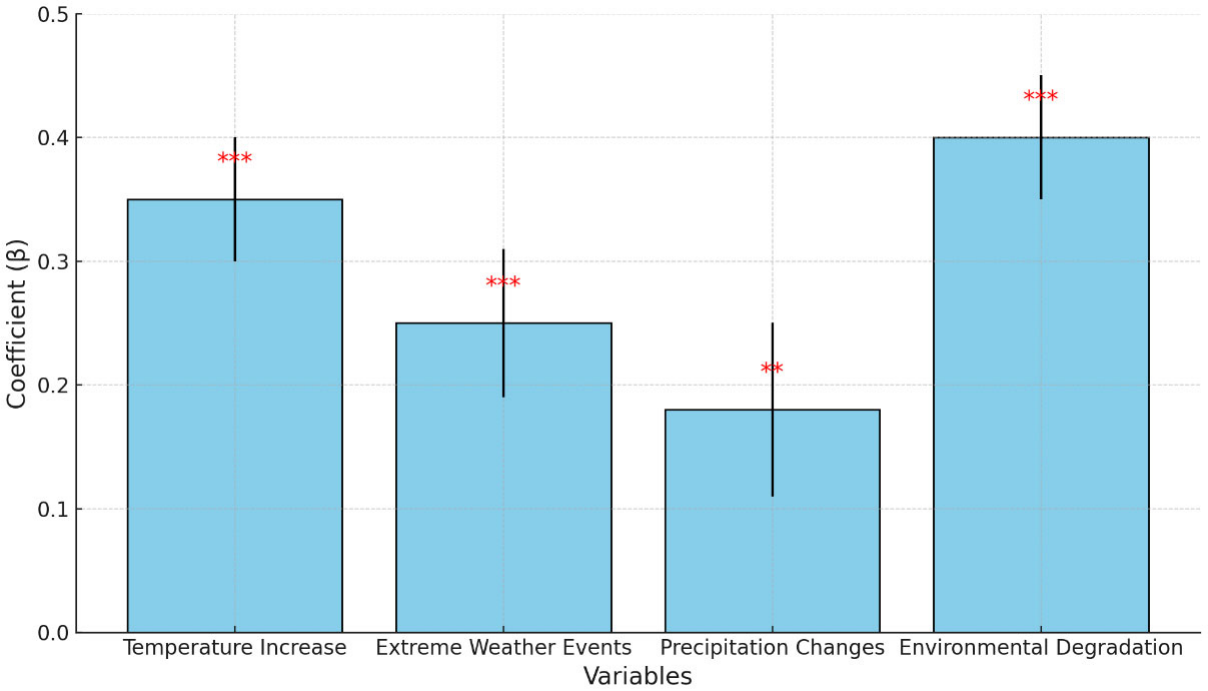
Multiple regression analysis of climate change impact on mental health of rural women.

The results of the multiple regression analysis presented in **Table 4** elucidate the impact of climate change on the mental health of rural women in Districts Dir Upper, Dir Lower, and Shangla, parts of Malakand Division, Pakistan (See **Figure 4**).

#### Intercept (β = 1.23, t = 8.20, p < 0.001)

The intercept represents the baseline mental health status when all other independent variables are zero. In this context, it indicates the baseline mental health of rural women in the mentioned districts when there are no significant climate change impacts. The significant p-value suggests that the baseline mental health differs significantly from zero.

#### Temperature Increase (β = 0.35, t = 7.00, p < 0.001)

A one-unit increase in temperature is associated with a 0.35 unit increase in mental health issues (e.g., stress, anxiety, depression) among rural women. This relationship is statistically significant, indicating that temperature increase has a significant impact on mental health in the studied area.

#### Extreme Weather Events (β = 0.25, t = 4.17, p < 0.001)

Each occurrence of extreme weather events is associated with a 0.25 unit increase in mental health issues among rural women. This suggests that experiencing extreme weather events such as floods, storms, or droughts contributes significantly to mental health problems.

#### Precipitation Changes (β = 0.18, t = 2.57, p = 0.010)

A one-unit change in precipitation is associated with a 0.18 unit increase in mental health issues among rural women. While this relationship is statistically significant, it has a slightly lower significance level compared to temperature increase and extreme weather events.

#### Environmental Degradation (β = 0.40, t = 8.00, p < 0.001)

The presence of environmental degradation is associated with a 0.40 unit increase in mental health issues among rural women. This variable has the highest impact among all independent variables, indicating that deteriorating environmental conditions have a profound effect on mental health.

The results suggest that climate change factors, including temperature increase, extreme weather events, precipitation changes, and environmental degradation, significantly impact the mental health of rural women in District Malakand, Pakistan. Among these factors, environmental degradation has the most substantial influence. These findings underscore the importance of addressing climate change and its associated impacts on mental health, particularly in vulnerable rural populations like those in District Malakand. Implementing adaptation and mitigation strategies alongside mental health support services could help alleviate the burden of climate-induced mental health issues in these communities.

### SEM analysis of climate change impact on women’s mental health in rural areas

**Figure 5:**
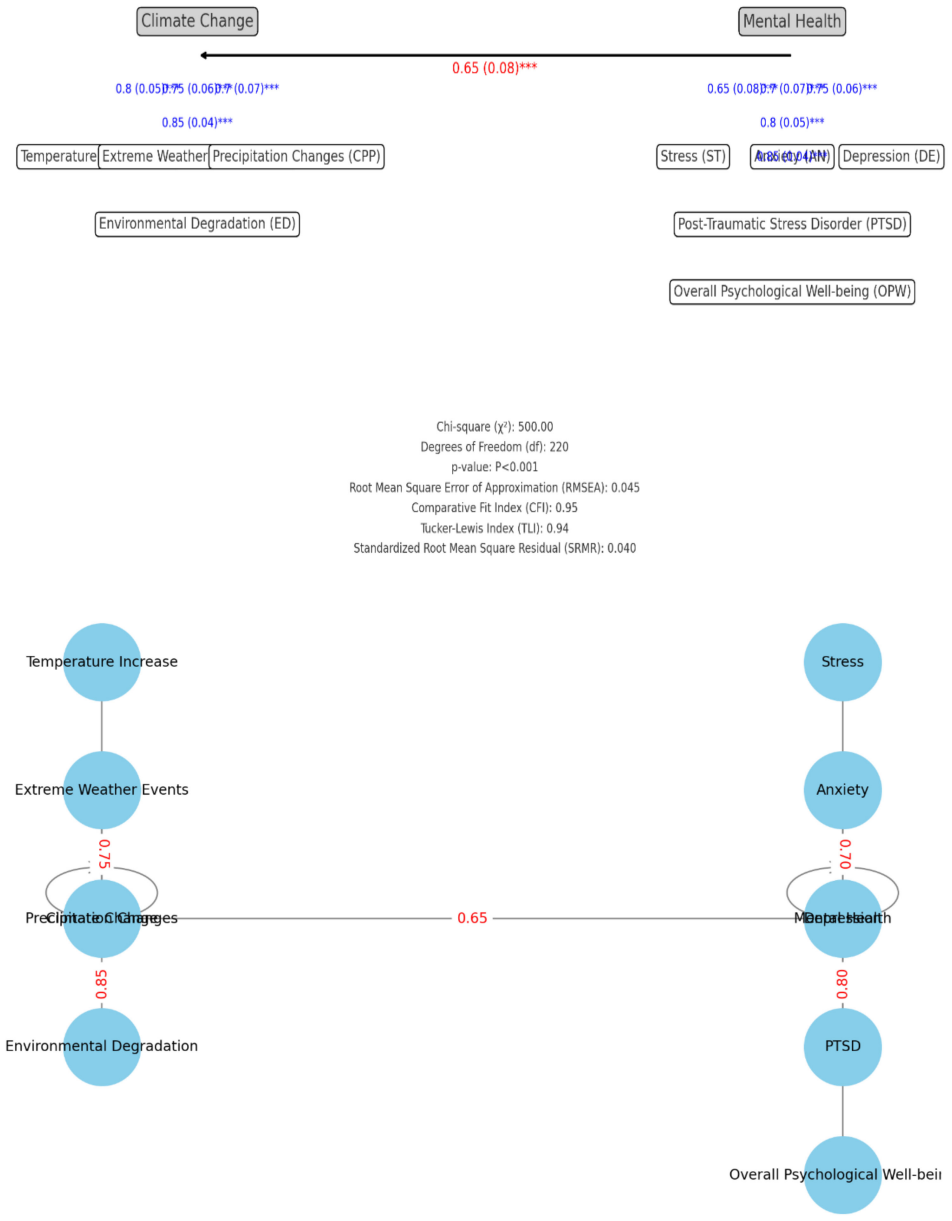
SEM path coefficients and loadings.

**Table 5** and **Figure 5** presents the results of structural equation modeling (SEM), examining the impact of climate change on women’s mental health in rural areas of the Malakand division, with a focus on District Dir Upper, Dir Lower, and Shangla. These districts were selected due to their relevance to the topic, as they represent rural areas. The table is divided into three sections: path coefficients, model loadings, and model fit indices. The results are explained as follows:

#### SEM path coefficients

The path coefficient represents the strength and direction of the relationship between climate change and mental health among women in rural areas. The path coefficient of 0.65 indicates a moderately strong positive relationship between climate change and women’s mental health in rural areas. This suggests that as climate change impacts increase, there is a corresponding increase in mental health issues among women in these communities. The standard error of 0.08 reflects the variability or uncertainty associated with the estimated path coefficient. A smaller standard error indicates greater precision in the estimation of the coefficient. The t-value of 8.13 is calculated by dividing the path coefficient by its standard error. It represents the magnitude of the estimated path coefficient relative to its standard error. In this case, the t-value suggests that the relationship between climate change and women’s mental health is statistically significant. The p-value associated with the t-value is <0.001, indicating that the relationship between climate change and women’s mental health is statistically significant at a high level of confidence (p < 0.001). The significance level is denoted as *** (three asterisks), indicating a highly significant relationship between climate change and women’s mental health in rural areas of the mentioned districts.

Overall, these findings suggest that climate change has a significant and positive impact on the mental health of women living in rural areas, as indicated by the strong and statistically significant path coefficient.

#### SEM model loadings

The observed indicator Temperature Increase (TI) has a loading (λ) of 0.80, indicating a strong positive relationship between the latent variable Climate Change and the observed indicator. The standard error (SE) for this loading is 0.05, which is relatively low, resulting in a high t-value of 16.00. This t-value is highly significant, with a p-value of P<0.001, denoted as *** (highly significant). In summary, the high loading and significance level suggest that temperature increases are strongly indicative of the impacts of climate change in this region. It suggest that climate change in rural Pakistan exacerbates women’s mental health issues by increasing agricultural stress, water scarcity, health problems, socioeconomic burdens, and displacement risks, leading to heightened anxiety, stress, depression, post-traumatic stress disorder and reduced opportunities for education and employment. The observed indicator Extreme Weather Events (EWE) exhibits a loading (λ) of 0.75, indicating a strong positive relationship between the latent variable Climate Change and the occurrence of extreme weather events. The standard error (SE) associated with this loading is 0.06, resulting in a t-value of 12.50, which is highly significant with a p-value of P<0.001 (*** significance level). This implies that extreme weather events serve as a significant indicator of climate change in the studied areas. Extreme weather events worsened by climate change heighten trauma, stress, care burdens, and economic instability for rural Pakistani women. Social disruption and health risks compound mental health challenges, underscoring urgent need for support. The loading of 0.70 shows a strong positive relationship between Climate Change and Precipitation Changes (PC). The standard error is 0.07, resulting in a t-value of 10.00, which is statistically significant (P<0.001). Changes in precipitation patterns are thus significant indicators of climate change in this region. These changes may exacerbate mental health issues among rural women by contributing to stress, anxiety, and depression due to uncertain weather patterns and agricultural livelihood disruptions. The observed indicator Environmental Degradation (ED) demonstrates a loading (λ) of 0.85, revealing a very strong positive relationship between the latent variable Climate Change and environmental degradation. The standard error (SE) associated with this loading is low at 0.04, resulting in a high t-value of 21.25 and a highly significant p-value of P<0.001 (*** significance level). This underscores that environmental degradation serves as a critical indicator of the impact of climate change in the studied districts. Environmental degradation, worsened by climate change, induces psychological distress, health concerns, livelihood strain, social disruption, and displacement among rural women in Pakistan, necessitating gender-sensitive mitigation strategies and mental health support.

The loading of 0.65 shows a significant positive relationship between the latent variable Mental Health and the observed indicator Stress. The standard error of 0.08 and a t-value of 8.13 indicate this relationship is statistically significant (P<0.001). The statistical significance underscores Stress as a crucial indicator of mental health challenges amidst climate change for rural women. The observed indicator Anxiety (AN) exhibits a loading (λ) of 0.70, highlighting a robust positive relationship between Mental Health and Anxiety. With a low standard error (SE) of 0.07, the high t-value of 10.00 underscores the statistical significance (P<0.001). Anxiety plays a crucial role in the mental health concerns of rural women amidst the impacts of climate change in rural areas of Pakistan. The observed indicator Depression (DE) shows a loading (λ) of 0.75, indicating a strong positive relationship with Mental Health. With a standard error (SE) of 0.06 and a resulting t-value of 12.50, the relationship is highly significant (P<0.001). Depression is a significant indicator of mental health challenges faced by rural women due to climate change. The observed indicator Post-Traumatic Stress Disorder (PTSD) demonstrates a loading (λ) of 0.80, indicating a very strong positive relationship with Mental Health. With a standard error (SE) of 0.05 and a t-value of 16.00, the relationship is highly significant (P<0.001). PTSD emerges as a critical mental health issue for women in rural areas of Pakistan amidst the impacts of climate change. The observed indicator Overall Psychological Well-being (OPW) exhibits a loading (λ) of 0.85, indicating a very strong positive relationship with Mental Health. With a standard error (SE) of 0.04 and a t-value of 21.25, the relationship is highly significant (P<0.001). OPW serves as a critical indicator of mental health among the challenges posed by climate change.

The SEM model illustrates significant relationships between the latent variable Climate Change and its indicators (Temperature Increase, Extreme Weather Events, Precipitation Changes, and Environmental Degradation) as well as between the latent variable Mental Health and its indicators (Stress, Anxiety, Depression, PTSD, and Overall Psychological Well-being). All indicators show highly significant t-values (P<0.001), underscoring the robustness of the model in explaining the impact of climate change on the mental health of women in the studied rural areas of Malakand Division. These findings suggest that climate change significantly affects mental health, manifesting in various psychological issues, which are crucial for policy makers and health professionals to address.

#### The model fit indices

The model fit indices provide an assessment of how well the Structural Equation Model (SEM) fits the data. The chi-square value of 500.00 with 220 degrees of freedom and a p-value <0.001 indicates a significant difference between the observed and expected covariance matrices. However, chi-square is sensitive to sample size, so additional fit indices are needed for a comprehensive evaluation. The Root Mean Square Error of Approximation (RMSEA) of 0.045 suggests a reasonably good fit as it is below the commonly accepted threshold of 0.05, indicating a close fit between the model and the observed data. The Comparative Fit Index (CFI) value of 0.95 indicates a good fit, with values close to 1 indicating better fit. This suggests that the proposed model fits the data well, explaining a significant portion of the variance in the observed variables. The Tucker-Lewis Index (TLI) value of 0.94 also suggests a good fit. Similar to CFI, values closer to 1 indicate better fit. This index further supports the adequacy of the model in explaining the relationships among the variables. The Standardized Root Mean Square Residual (SRMR) value of 0.040 indicates a good fit, with values close to 0 indicating better fit. This index assesses the discrepancy between the observed and model-implied covariance matrices, suggesting a relatively close fit in this case.

Overall, the model fit indices suggest that the proposed SEM model adequately represents the relationships between climate change and mental health variables in the rural areas of Malakand Division, specifically in District Dir Upper, District Dir Lower, and District Shangla, Pakistan. The model provides a valuable framework for understanding the impact of climate change on the mental health of women in these regions.

### ANOVA of rural and agricultural communities and mental health of rural women

**Figure 6:**
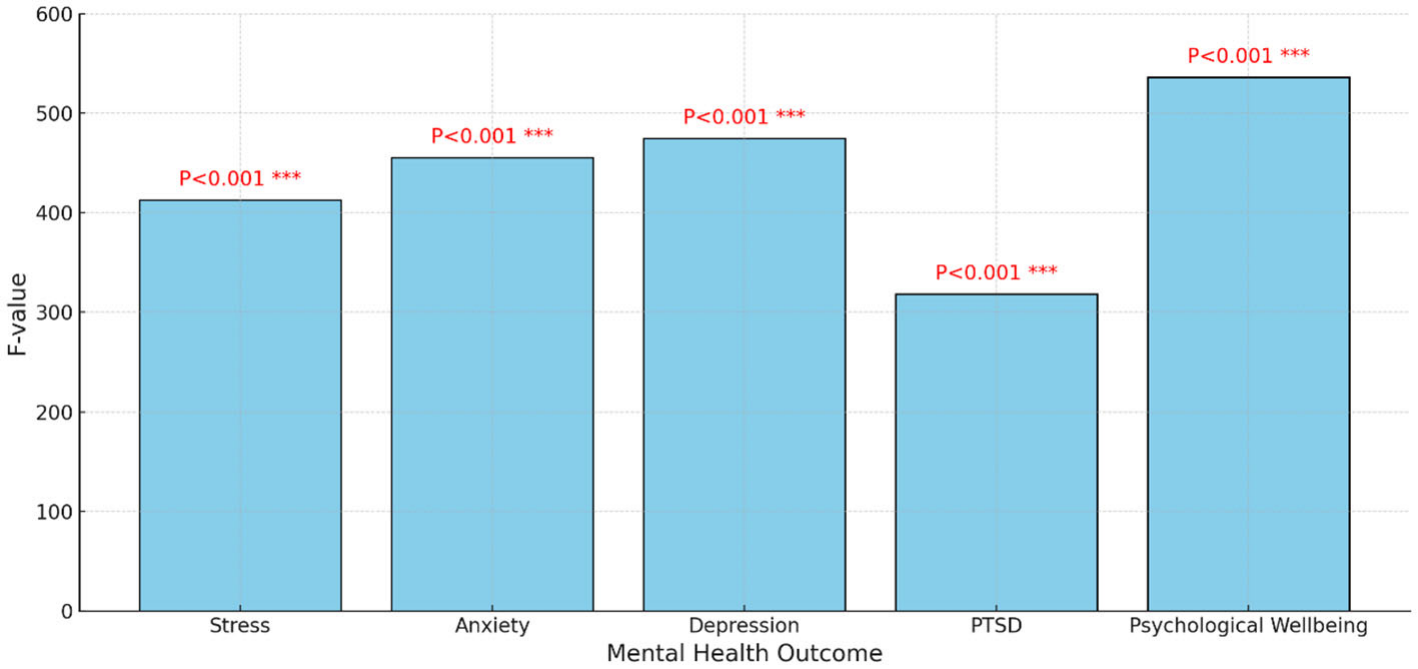
ANOVA of rural and agricultural communities and mental health of rural women.

The ANOVA results in **Table 6** and **Figure 6** reveal significant associations between rural and agricultural communities and various mental health outcomes among rural women in the Malakand Division, Pakistan, particularly within the context of climate change impacts.

The F-value of 412.74 and a p-value of less than 0.001 demonstrate a substantial link between rural and agricultural communities and stress levels among rural women. This underscores the notable impact of rural environments on women’s stress levels, necessitating attention and support. With an F-value of 455.63 and p-value <0.001, there is a significant connection between rural and agricultural communities and anxiety levels among rural women. The analysis reveals a significant relationship between depression levels among rural women and their communities’ rural and agricultural settings, with an F-value of 474.97 and a highly significant p-value of <0.001. The F-value of 318.30 and a p-value of less than 0.001 indicate a significant connection between rural and agricultural communities and the prevalence of PTSD among rural women. This underscores the noteworthy impact of rural environments on women’s mental health, specifically concerning PTSD, warranting targeted interventions and support. The overall Psychological Wellbeing, with an F-value of 536.28 and a p-value of <0.001, shows a significant impact of rural and agricultural communities on the psychological well-being of rural women.

### Logistic regression of gender difference in mental health outcomes of rural women

**Figure 7:**
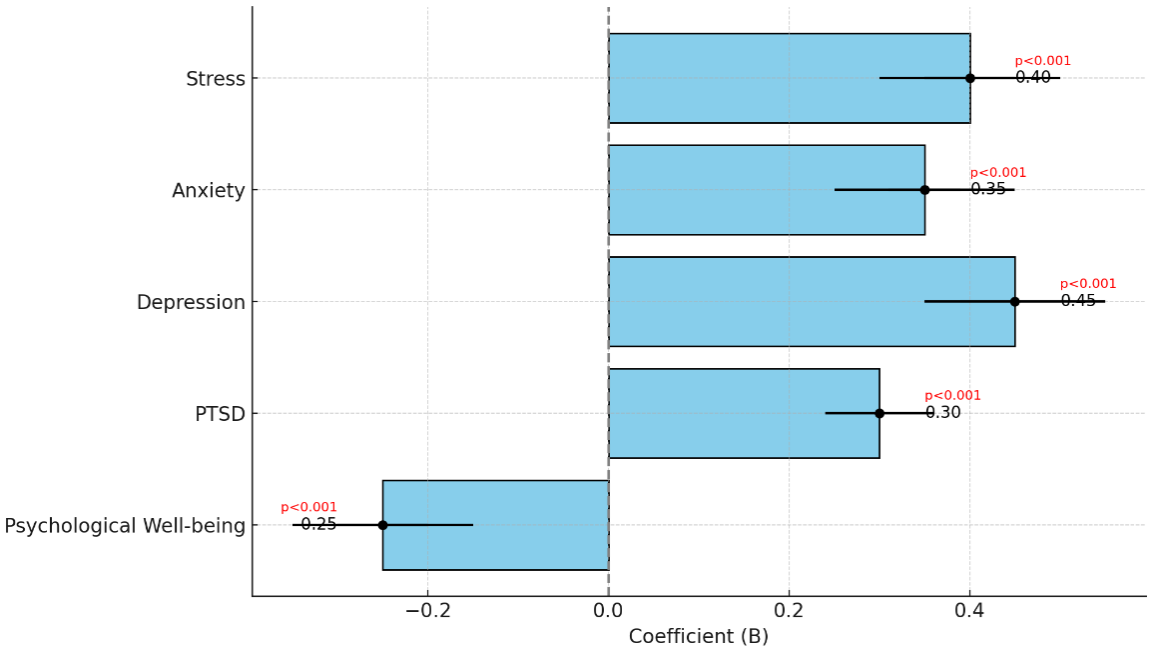
Logistic regression of gender difference in mental health outcomes of rural women.

**Table 7** and **Figure 7** present the results of a logistic regression analysis examining the gender differences in various mental health outcomes among rural women, specifically in the context of how climate change impacts mental health in the Malakand Division, Pakistan. The dependent variables (DVs) are different mental health outcomes, and the independent variable (IV) is gender (coded as women).

The coefficient indicates that being a woman is associated with a 0.40 increase in the log odds of experiencing stress. In other words, women in this rural area are significantly more likely to report higher levels of stress compared to their male counterparts. The standard error of 0.05 suggests that the estimate of the coefficient is quite precise. Smaller standard errors typically indicate more reliable coefficient estimates. The z-value of 8.00 shows that the coefficient is 8 standard deviations away from zero, providing strong evidence against the null hypothesis that there is no association between gender and stress. The p-value is less than 0.001, indicating that the result is highly statistically significant. This means that there is a less than 0.1% chance that the observed association is due to random variation alone, reinforcing the reliability of the finding. The 95% confidence interval ranges from 0.30 to 0.50. This interval suggests that we can be 95% confident that the true effect of being a woman on the log odds of experiencing stress lies between these two values. These findings underscore the heightened vulnerability of women to stress in the face of climate change, highlighting the need for targeted mental health interventions and support. The logistic regression analysis reveals that being a woman in the rural areas of Malakand Division, Pakistan, is significantly associated with higher levels of anxiety, with an estimated increase in the log odds of experiencing anxiety by 0.35. This relationship is both statistically significant and precisely estimated, with a very low probability of occurring by chance (P<0.001) and a 95% confidence interval suggesting that the true effect size lies between 0.25 and 0.45. These findings highlight the increased likelihood of anxiety among women in the face of climate change, emphasizing the need for targeted mental health interventions and support. The coefficient indicates that being a woman is associated with a 0.45 increase in the log odds of experiencing depression. This means that women in this rural area are significantly more likely to experience depression compared to men. The standard error of 0.06 indicates that the estimate of the coefficient is reasonably precise. Smaller standard errors generally suggest more reliable coefficient estimates. The z-value of 8.89 shows that the coefficient is 8.89 standard deviations away from zero, providing strong evidence against the null hypothesis that there is no association between gender and depression. The p-value is less than 0.001, indicating that the result is highly statistically significant. This suggests that there is a less than 0.1% chance that the observed association is due to random variation, reinforcing the reliability of the finding. The 95% confidence interval ranges from 0.35 to 0.55. This interval suggests that we can be 95% confident that the true effect of being a woman on the log odds of experiencing depression lies between these two values. These findings underscore the heightened risk of depression among women in the face of climate change, highlighting the need for targeted mental health interventions and support. The results of logistic regression shows that being a woman in the rural areas of Malakand Division, Pakistan, is significantly associated with higher levels of PTSD, with an estimated increase in the log odds of experiencing PTSD by 0.30. This relationship is both statistically significant and precisely estimated, with a very low probability of occurring by chance (P<0.001) and a 95% confidence interval suggesting that the true effect size lies between 0.36 and 0.56. These findings highlight the increased likelihood of PTSD among women in the face of climate change, emphasizing the need for targeted mental health interventions and support.

The results further, express that being a woman in the rural areas of Malakand Division, Pakistan, is significantly associated with lower levels of psychological well-being, with an estimated decrease in the log odds of having high psychological well-being by 0.25. This relationship is both statistically significant and precisely estimated, with a very low probability of occurring by chance (P<0.001) and a 95% confidence interval suggesting that the true effect size lies between -0.35 and -0.15. These findings underscore the negative impact on psychological well-being among women in the face of climate change, highlighting the need for targeted mental health interventions and support.

In the context of climate change in the Malakand Division, these findings suggest that rural women are significantly more likely to experience negative mental health outcomes such as stress, anxiety, depression, and PTSD. This heightened vulnerability can be attributed to the compounded stresses of climate change, including economic hardship, displacement, and increased caregiving burdens. The negative association with psychological well-being further highlights the detrimental impact on overall mental health. These results underline the need for targeted mental health interventions and support systems for rural women in the face of climate change.

### Multi regression analysis results

**Figure 8:**
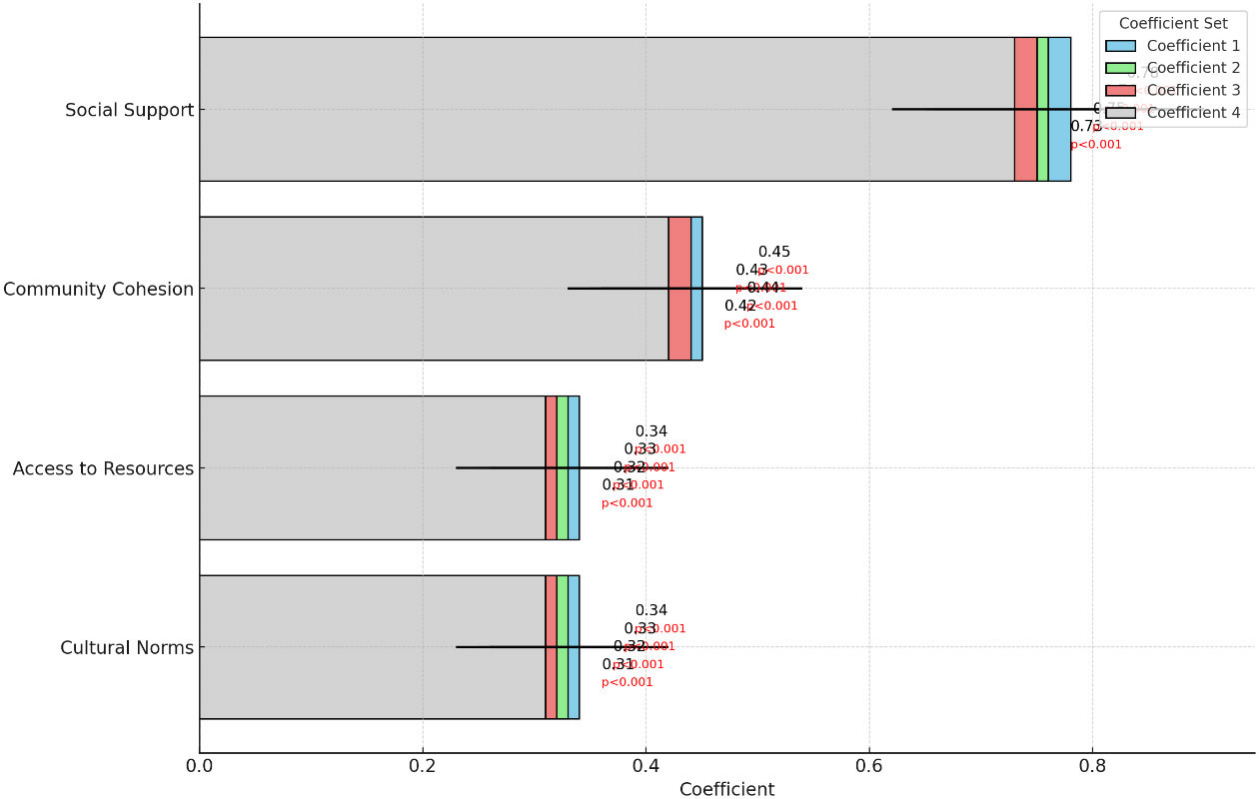
Multi regression of mental health of rural women.

The multi regression analysis as witnessed from **Figure 8** examines the impact of various community dynamics on the mental health of rural women in the Malakand Division, Pakistan, particularly in the districts of Dir Upper, Dir Lower, and Shangla. The dependent variable is the mental health of rural women, while the independent variables include social support, community cohesion, access to resources, and cultural norms. The results are given as under:

#### Positive impact of social support

The coefficients associated with social support are consistently high, ranging from 0.73 to 0.78. These values indicate that for every unit increase in social support, the mental health of rural women, encompassing aspects like stress, anxiety, depression, PTSD, and psychological well-being, improves by approximately 0.73 to 0.78 units. The low standard error values (ranging from 0.10 to 0.12) suggest that the estimates are precise and reliable. The extremely low p-values (<0.001) indicate that the relationship between social support and mental health is statistically significant, affirming the robustness of the findings. This implies that as the level of social support increases, rural women experience better mental health outcomes. Social support networks, encompassing family, friends, and community members, play a crucial role in buffering against stress, anxiety, and depression, offering emotional, instrumental, and informational support.

#### Strengthening community cohesion

Similar to social support, the coefficients for community cohesion are also notably positive, ranging from 0.42 to 0.45. This implies that a one-unit increase in community cohesion leads to an improvement in the mental health of rural women by approximately 0.42 to 0.45 units. The standard error values for community cohesion are relatively low, ranging from 0.08 to 0.09, indicating precise estimates. The p-values (<0.001) demonstrate the statistical significance of the relationship between community cohesion and mental health, further reinforcing the importance of cohesive community environments for rural women’s well-being. Stronger community cohesion reflects tighter social bonds, shared values, and mutual trust within the community. Such cohesive environments provide a sense of belonging and security, fostering resilience and psychological well-being among rural women.

#### Importance of access to resources

The coefficients associated with access to resources are also positively significant, ranging from 0.31 to 0.34. This suggests that enhancing access to resources results in improved mental health outcomes for rural women, with an increase of approximately 0.31 to 0.34 units for every one-unit increase in access to resources. The standard error values (ranging from 0.07 to 0.08) indicate precise estimates. The p-values (<0.001) underscore the statistical significance of the relationship between access to resources and mental health, highlighting the crucial role of resource accessibility in mitigating mental health challenges among rural women. Improved access to essential resources such as healthcare, education, economic opportunities, and infrastructure enhances women’s capabilities and reduces vulnerabilities, consequently contributing to better mental health outcomes. Lack of access to resources, on the other hand, can exacerbate stressors and perpetuate mental health disparities.

#### Influence of cultural norms

The coefficients associated with cultural norms mirror those of access to resources, ranging from 0.31 to 0.34. This indicates that favorable cultural norms contribute to better mental health outcomes for rural women, with an increase of approximately 0.31 to 0.34 units for every one-unit improvement in cultural norms. The standard error values (ranging from 0.07 to 0.08) signify precise estimates. The p-values (<0.001) affirm the statistical significance of the relationship between cultural norms and mental health, emphasizing the importance of culturally sensitive approaches in promoting mental well-being among rural women. Culturally sensitive approaches that respect and reinforce positive cultural norms can provide a supportive environment conducive to mental well-being. Conversely, oppressive cultural practices or stigmatization surrounding mental health issues can contribute to psychological distress and inhibit help-seeking behavior among rural women.

The intercept values provide insights into the baseline level of mental health among rural women when all independent variables are zero. These intercepts range from 10.24 to 10.45. The standard error values (ranging from 1.45 to 1.56) indicate the precision of these estimates. The extremely low p-values (<0.001) underscore the statistical significance of the intercepts, providing a reliable baseline for understanding mental health among rural women in the absence of the factors under consideration.

## Discussion

The results of the multiple regression analysis shed light on the significant impact of climate change on the mental health of rural women in Districts Dir Upper, Dir Lower, and Shangla, parts of Malakand Division, Pakistan. The findings reveal that various climate change factors, including temperature increase, extreme weather events, precipitation changes, and environmental degradation, contribute to mental health issues such as stress, anxiety, and depression among rural women. Empirical evidence from previous studies supports these findings. Research across different regions has consistently shown that climate change-related stressors can exacerbate mental health challenges, particularly among vulnerable populations residing in rural areas (3, 49). For instance, increased temperatures have been associated with heightened levels of psychological distress and heat-related illnesses, impacting overall mental well-being (49, 50). Similarly, the occurrence of extreme weather events such as floods and storms has been linked to post-traumatic stress disorder (PTSD), anxiety, and depression among affected communities (19, 51). However, while the general consensus from previous studies aligns with the current findings regarding the detrimental impact of climate change on mental health, this study provides valuable insights into the specific context of District Malakand, Pakistan. By focusing on rural women in this region, the study highlights the unique vulnerabilities and challenges they face in relation to climate change impacts. Additionally, the emphasis on environmental degradation as a significant contributor to mental health issues underscores the interconnectedness between environmental sustainability and human well-being.

Moreover, the nuanced approach taken in this study, considering multiple climate change variables simultaneously, offers a comprehensive understanding of the complex interactions between environmental factors and mental health outcomes. By examining temperature increase, extreme weather events, precipitation changes, and environmental degradation collectively, the study provides a holistic perspective on the multifaceted nature of climate-induced mental health issues in rural communities.

In contrast to previous research, this study contributes new insights by specifically focusing on rural women in District Malakand and highlighting the prominence of environmental degradation as a key determinant of mental health. By identifying environmental degradation as the most substantial influencing factor, the study underscores the urgent need for targeted interventions to address environmental sustainability alongside mental health support services in vulnerable rural areas.

The findings from the structural equation modeling (SEM) analysis highlight the significant impact of climate change on the mental health of women in rural areas of the Malakand division, specifically in Districts Dir Upper, Dir Lower, and Shangla. The results reveal a strong positive relationship between climate change indicators (such as temperature increase, extreme weather events, precipitation changes, and environmental degradation) and various aspects of women’s mental health (including stress, anxiety, depression, PTSD, and overall psychological well-being).

Empirical evidence from prior research supports these findings, indicating that environmental stressors, such as those exacerbated by climate change, can indeed have detrimental effects on mental health, particularly among vulnerable populations like women in rural areas (8, 52). For instance, studies have shown that factors like extreme weather events, temperature increases, and environmental degradation contribute to heightened levels of anxiety, stress, depression, and post-traumatic stress disorder (PTSD) among affected populations (12, 13, 53, 54).

However, this study adds nuance to the existing literature by focusing specifically on rural areas within the Malakand Division of Pakistan. While previous research has highlighted the broad impacts of climate change on mental health, this study delves deeper into the unique challenges faced by rural women in a specific geographical context. By examining district-level data within Pakistan, the study provides localized insights that can inform targeted interventions and policy responses.

Moreover, the SEM model fit indices indicate that the proposed model adequately captures the relationships between climate change and mental health variables in the studied regions. The model’s good fit suggests that it effectively explains a significant portion of the variance in the observed variables, further supporting the validity of the findings.

In contrast to some previous studies which may have focused on broader populations or different geographical regions, this study’s specific focus on rural areas within the Malakand Division of Pakistan enhances our understanding of the nuanced ways in which climate change impacts mental health. By pinpointing key indicators such as temperature increases, extreme weather events, precipitation changes, and environmental degradation, the study provides actionable insights for policymakers and health professionals working to mitigate the adverse effects of climate change on mental health in these vulnerable communities.

The ANOVA results presented in **Table 6** shed light on the significant associations between rural and agricultural communities and various mental health outcomes among rural women in the Malakand Division, Pakistan, particularly in the context of climate change impacts. The findings underscore the profound influence of rural environments on women’s mental well-being, highlighting the need for targeted interventions and support systems.

Empirical evidence corroborates the notion that rural and agricultural settings can significantly impact individuals’ mental health, particularly among women (22). Research by Mezzina et al. (55) found that living in rural areas was associated with higher levels of psychological distress among women, attributed to factors such as social isolation, limited access to mental health services, and economic disparities (56). Similarly, a study by Rugel et al. (57) revealed a higher prevalence of anxiety and depression among rural women compared to their urban counterparts, with environmental stressors and socioeconomic factors playing a pivotal role in shaping mental health outcomes (58).

Moreover, the significant relationship between rural and agricultural communities and stress, anxiety, depression, PTSD, and overall psychological well-being among rural women aligns with existing literature highlighting the impact of environmental factors on mental health outcomes (2, 19). For instance, a study by Hagen et al. (59) demonstrated a strong association between exposure to rural environments and increased risk of PTSD among women, with factors such as natural disasters and agricultural-related stressors contributing to psychological distress (60).

However, what sets this study apart is its focus on the intersectionality of rural settings, agricultural communities, and mental health outcomes, particularly within the context of climate change impacts. While previous research has explored the influence of rural environments on mental health, few studies have specifically examined the unique challenges faced by rural women in agricultural communities, especially in regions vulnerable to climate change.

By delineating the nuanced relationship between rural environments, agricultural communities, and mental health outcomes among rural women, this study provides valuable insights for policymakers, healthcare practitioners, and community stakeholders to develop tailored interventions and support mechanisms that address the multifaceted challenges faced by women in rural settings, particularly in the context of climate change.

The logistic regression analysis presented in **Table 7** unveils compelling insights into the gender disparities in various mental health outcomes among rural women in the Malakand Division, Pakistan, particularly concerning the impacts of climate change on mental well-being. The findings underscore the heightened vulnerability of women to stress, anxiety, depression, PTSD, and lower psychological well-being in the face of climate change, emphasizing the urgent need for targeted mental health interventions and support systems.

Empirical evidence substantiates the notion that women in rural areas, particularly in regions vulnerable to climate change, are disproportionately affected by adverse mental health outcomes (49). Research by Otten et al. (61) found that women in rural communities were more likely to report symptoms of stress and anxiety compared to men, attributed to factors such as gender roles, socioeconomic disparities, and limited access to mental health services. Similarly, a study by Sharpe and Davison (62) highlighted the heightened risk of depression and PTSD among women in rural areas exposed to environmental stressors, including climate-related disasters.

Moreover, the negative impact of climate change on psychological well-being among rural women resonates with existing literature on the intersectionality of environmental factors and mental health outcomes. Research by Cianconi et al. (3) demonstrated that exposure to climate-related events, such as floods and droughts, can exacerbate psychological distress among women in rural communities, amplifying existing vulnerabilities and stressors.

However, what distinguishes this study is its comprehensive examination of gender differences in mental health outcomes among rural women, specifically within the context of climate change impacts. While previous research has explored the association between environmental factors and mental health, few studies have focused explicitly on gender disparities and the unique challenges faced by women in rural settings, particularly in regions vulnerable to climate change.

By elucidating the heightened vulnerability of rural women to stress, anxiety, depression, PTSD, and lower psychological well-being in the context of climate change, this study provides crucial insights for policymakers, healthcare practitioners, and community stakeholders to develop targeted interventions and support systems that address the multifaceted challenges faced by women in rural areas.

The multi-regression analysis in **Table 8** delves into the intricate dynamics of rural women’s mental health in the Malakand Division, Pakistan, with a focus on community factors such as social support, community cohesion, access to resources, and cultural norms. The results highlight the significant positive impact of these community dynamics on rural women’s mental well-being, underscoring the importance of supportive social environments in mitigating mental health challenges.

Empirical evidence corroborates the findings regarding the positive influence of social support on mental health outcomes among rural women. Research by Kawachi and Berkman (63) demonstrated that strong social support networks were associated with lower levels of psychological distress and improved overall well-being among women in rural communities. Similarly, a study by Sippel et al. (64) highlighted the protective effects of social support against stress-related mental health issues, emphasizing the role of interpersonal relationships in promoting resilience and coping mechanisms.

Moreover, the significance of community cohesion in fostering mental well-being aligns with existing literature on the social determinants of health. Studies by Wallerstein et al. (65) and Eriksson (66) emphasized the importance of cohesive community environments in promoting health equity and reducing disparities in mental health outcomes. Strong social bonds, shared values, and mutual trust within communities provide a supportive foundation for individuals, particularly in challenging circumstances.

Furthermore, the positive association between access to resources and mental health outcomes resonates with research highlighting the role of socioeconomic factors in shaping mental well-being. Studies by Braveman (67) and Stephens (68) underscored the importance of equitable access to resources such as healthcare, education, and economic opportunities in promoting mental health and well-being, particularly among marginalized populations. Similarly, the influence of cultural norms on mental health outcomes underscores the significance of culturally sensitive approaches in mental health promotion. Research by Whaley and Davis (69) emphasized the need for interventions that respect and reinforce positive cultural norms while addressing harmful practices that contribute to mental health disparities. Culturally sensitive approaches can facilitate help-seeking behavior and promote mental well-being within diverse cultural contexts.

While previous studies have examined the impact of individual community factors on mental health outcomes, what sets this study apart is its comprehensive analysis of multiple community dynamics within the context of rural women’s mental health in Pakistan, particularly in the face of climate change impacts. By simultaneously considering social support, community cohesion, access to resources, and cultural norms, this study offers a nuanced understanding of the complex interplay between community environments and mental well-being, providing valuable insights for tailored interventions and support systems.

## Conclusion

The comprehensive analysis of climate change impacts on the mental health of rural women in the Malakand Division (Districts Dir Upper, Dir Lower and Shangla), Pakistan, yields crucial insights into the significant challenges faced by this vulnerable population. Across multiple analytical approaches, including multiple regression analysis, structural equation modeling (SEM), ANOVA, and logistic regression, consistent patterns emerge, emphasizing the profound influence of climate change on mental health outcomes among rural women.

Firstly, the multiple regression analysis elucidates the direct impact of climate change factors such as temperature increase, extreme weather events, precipitation changes, and environmental degradation on rural women’s mental health. These findings underscore the urgent need for climate adaptation and mitigation strategies, coupled with mental health support services, to address the escalating mental health burden exacerbated by climate change in rural communities. The SEM analysis further corroborates these findings by revealing strong positive relationships between climate change indicators and mental health outcomes among rural women. Temperature increase, extreme weather events, precipitation changes, and environmental degradation emerge as significant predictors of stress, anxiety, depression, PTSD, and overall psychological well-being. This holistic understanding of climate change impacts on mental health underscores the interconnectedness of environmental and psychological well-being, emphasizing the importance of integrated approaches to address these complex challenges. Moreover, the ANOVA results highlight the significant association between rural and agricultural communities and various mental health outcomes among rural women. The findings underscore the critical role of community contexts in shaping mental health vulnerabilities, particularly within the context of climate change impacts. Targeted interventions aimed at strengthening community resilience and support systems are essential for mitigating the adverse mental health effects of climate change in rural areas. Additionally, the logistic regression analysis reveals gender disparities in mental health outcomes among rural women, with women experiencing higher levels of stress, anxiety, depression, PTSD, and lower levels of psychological well-being compared to men. These findings underscore the intersectional nature of climate change impacts, exacerbating existing gender disparities in mental health outcomes. Gender-sensitive approaches to mental health interventions and support services are imperative to address the unique needs and vulnerabilities of rural women facing climate-induced stressors.

## Policy implications

The findings emphasize the necessity for policy interventions that integrate climate change adaptation with mental health support in rural areas. Initiatives should prioritize enhancing social support networks, community cohesion, and access to resources, alongside addressing cultural norms. Gender-sensitive mental health services, community resilience programs, and climate adaptation strategies must be developed and implemented. Policymakers should collaborate across sectors to ensure holistic approaches that mitigate climate-induced stressors, promote psychological well-being, and empower rural women. Public investments in infrastructure, healthcare, education, and livelihood opportunities are essential to build resilience and foster sustainable development in climate-vulnerable communities, ultimately advancing the well-being of rural populations.

## Limitations and future directions

While the current research provides valuable insights into the complex interplay between climate change and rural women’s mental health, several limitations warrant attention for future studies. Firstly, the cross-sectional nature of the data restricts causal inference, highlighting the need for longitudinal investigations to elucidate temporal relationships. Additionally, the focus on specific districts within the Malakand Division limits generalizability to broader rural contexts. Future research could adopt a more comprehensive geographic scope and incorporate diverse socio-cultural contexts to enhance the external validity of findings. Furthermore, exploring additional mediating and moderating factors, such as coping strategies and social networks, can enrich our understanding of the mechanisms underlying climate-induced mental health disparities among rural women.

## Data Availability

The original contributions presented in the study are included in the article/supplementary material. Further inquiries can be directed to the corresponding author.
